# Novel Disease-Associated Missense Single-Nucleotide Polymorphisms Variants Predication by Algorithms Tools and Molecular Dynamics Simulation of Human TCIRG1 Gene Causing Congenital Neutropenia and Osteopetrosis

**DOI:** 10.3389/fmolb.2022.879875

**Published:** 2022-04-28

**Authors:** Khyber Shinwari, Hafiz Muzzammel Rehman, Guojun Liu, Mikhail A. Bolkov, Irina A. Tuzankina, Valery. A. Chereshnev

**Affiliations:** ^1^ Institute of Chemical Engineering, Department of Immunochemistry, Ural Federal University, Yekaterinburg, Russia; ^2^ School of Biochemistry and Biotechnology, University of the Punjab, Lahore, Pakistan; ^3^ Alnoorians Group of Institutes, Shad Bagh, Lahore, Pakistan; ^4^ School of Life Science and Technology, Inner Mongolia University of Science and Technology, Baotou, China; ^5^ Institute of Immunology and Physiology of the Ural Branch of the Russian Academy of Sciences, Yekaterinburg, Russia

**Keywords:** TCIRG1 gene mutation, congenital neutropenia, osteopetrosis, non-synonymous single nucleotide polymorphisms, molecular dynamics simulation (MD)

## Abstract

T Cell Immune Regulator 1, ATPase H + Transporting V0 Subunit A3 (TCIRG1 gene provides instructions for making one part, the a3 subunit, of a large protein complex known as a vacuolar H + -ATPase (V-ATPase). V-ATPases are a group of similar complexes that act as pumps to move positively charged hydrogen atoms (protons) across membranes. Single amino acid changes in highly conserved areas of the TCIRG1 protein have been linked to autosomal recessive osteopetrosis and severe congenital neutropenia. We used multiple computational approaches to classify disease-prone single nucleotide polymorphisms (SNPs) in TCIRG1. We used molecular dynamics analysis to identify the deleterious nsSNPs, build mutant protein structures, and assess the impact of mutation. Our results show that fifteen nsSNPs (rs199902030, rs200149541, rs372499913, rs267605221, rs374941368, rs375717418, rs80008675, rs149792489, rs116675104, rs121908250, rs121908251, rs121908251, rs149792489 and rs116675104) variants are likely to be highly deleterious mutations as by incorporating them into wild protein they destabilize the wild protein structure and function. They are also located in the V-ATPase I domain, which may destabilize the structure and impair TCIRG1 protein activation, as well as reduce its ATPase effectiveness. These mutants have not yet been identified in patients suffering from CN and osteopetrosis while (G405R, R444L, and D517N) reported in our study are already associated with osteopetrosis. Mutation V52L reported in our study was identified in a patient suspected for CN. Finally, these mutants can help to further understand the broad pool of illness susceptibilities associated with TCIRG1 catalytic kinase domain activation and aid in the development of an effective treatment for associated diseases.

## 1 Introduction

A precise balance between bone creation by osteoblasts and resorption by osteoclasts is required for bone development and homeostasis. Osteopetrosis is a hereditary disease defined by a clinically and genetically heterogeneous group of bone resorption diseases. The three primary types based on hereditary patterns are the age of onset, severity, and type (Tolar et al., 2004). All of these variants have increased bone density, which can cause fractures, osteomyelitis, deformity, dental anomalies, bone marrow failure, and cranial nerve compression, among other phenotypical features (Stark and Savarirayan, 2009). Osteopetrosis is a rare condition, occurring in about one in every 250,000 births, compared to one in every 20,000 births for autosomal dominant osteopetrosis (Loría-Cortés et al., 1977). These conditions are more common in some geographical places, such as Costa Rica, the Middle East, Russia’s Chuvash Republic, and Northern Sweden’s Västerbotten Province. This spread is aided by the founder effect, geographical isolation, and severe maternal consanguinity (Sobacchi et al., 2013a). Numerous forms of osteopetrosis cases in humans have been linked to changes in at least ten genes (Stark and Savarirayan, 2009). Autosomal recessive osteopetrosis renders bones more sensitive to hematological damage and neurological deficit as a result of a smaller bone marrow cavity and nerve compression (blindness or deafness). In a study published in 2000, changes in T Cell Immune Regulator 1, ATPase H + Transporting V0 Subunit A3 were identified to be a primary source of human autosomal recessive osteopetrosis (Sobacchi et al., 2013a). As a result of the molecular analysis, six new genes (TNFSF11, TNFRSF11A, CLCN7, OSTM1, SNX10, and PLEKHM1) have been discovered to be associated with human ARO. More than half of all autosomal recessive osteopetrosis patients had TCIRG1 mutations (Sobacchi et al., 2001). According to a study, mice with a targeted disruption of Atp6i developed severe osteopetrosis (Li et al., 1999). Despite tremendous progress in our understanding of disease mechanisms in osteoporotic diseases, the genetic basis for 30% of cases is unclear (Sobacchi et al., 2013b). According to the study, TCIRG1 mutations include missense, nonsense, small deletions/insertions, splice-site mutations, significant genomic deletions, and intronic mutations (Frattini et al., 2000; Kornak et al., 2000; Sobacchi et al., 2013b; Sobacchi et al., 2014; Palagano et al., 2015). There is still a link between autosomal recessive osteopetrosis 1 and premature infertility deaths. This issue can be detected as early as the age of 10 days. The most prevalent signs of the illness are pathologic fractures, bone marrow failure, and cranial nerve compression, which are caused by impaired bone turnover, metabolism, and failure to widen cranial nerve foramina (Chávez-Güitrón et al., 2018). High bone density can occur from a bone resorption fault caused by osteoclast dysfunction, which can lead to severe abnormalities. Some of the defects that appear early in fetal development include microcephaly, progressive deafness, blindness, hepatosplenomegaly, and severe anemia. Deafness and blindness are common side effects of secondary cranial nerve hypertension (Susani et al., 2004). Sever Congenital Neutropenia is a hematological condition characterized by low blood neutrophil counts (ANC) of less than 0.5 109/L and recurrent bacterial infections that usually start in childhood. In 1956, Kostmann was the first to describe an autosomal recessive form of sever congenital neutropenia (KOSTMANN, 1956). A recessive type of sever congenital neutropenia is considered to be caused by mutations in HAX1, a gene related to the Bcl-2 family (Carlsson and Fasth, 2001; Klein et al., 2007). Mutations in the ELA2 gene, which codes for the protein neutrophil elastase, an enzyme present in the major granules of neutrophils, are the most common cause of sever congenital neutropenia (Horwitz et al., 1999; Dale et al., 2000). Other genes which can induce neutropenia, include such as those involved in glucose homeostasis (SLC37A4, G6PC3), lysosomal function (LYST, RAB27A, ROBLD3/p14, AP3B1, VPS13B), ribosomal proteins (SBDS, RMRP), mitochondrial proteins (HAX1, AK2, TAZ), immunological functions (STK4, GFI1, CXCR4), and Xlinked (WAS) (Boztug and Klein, 2009). In contrast, many families with autosomal dominant sever congenital neutropenia have no identifiable mutation, showing that there are more sever congenital neutropenia genes. After high-density SNP chips were used to detect IBD regions across affected in a large SCN family, exome sequencing was utilized to find coding single nucleotide variants (SNVs) in the IBD regions (Makaryan et al., 2014). SNPs (single nucleotide polymorphisms) are genetic markers found in the human genome at each 200–300 base pair (Lee et al., 2005). There are roughly 0.5 million SNPs in the human genome’s coding region (Rajasekaran et al., 2008). Substituting amino acids is conserved areas can change the structure, stability, and function of proteins. Nonsynonymous SNPs (nsSNPs) are known to alter protein function and have a higher chance of causing disease in humans (George Priya Doss et al., 2008; Chitrala and Yeguvapalli, 2014; Shinwari et al., 2021). Evidently, several studies have shown that nsSNPs are responsible for 50% of the variations related to heredity genetic disorders (Ramensky et al., 2002; Doniger et al., 2008; Radivojac et al., 2010). Alignment methods based on matrix and data tree structure computation are being used by the instruments (Kamatani et al., 2004; Rajasekaran et al., 2008). We described the structural and functional impacts of high-risk nsSNPs on the TCIRG1 protein using a series of prediction algorithms.

## 2 Methods

### 2.1 SNP Retrieval

The nsSNP information for the human TCIRG1 gene was obtained utilizing a variety of web-based data sources, including OMIM (Online Mendelian Inheritance in Man) (Hamosh et al., 2005), NCBI dbSNP (Sherry et al., 2001), and the UniProt database (UniProtKB ID O15072) (UniProt Consortium, 2010).

### 2.2 Gene Mania

Gene MANIA (https://genemania.org/) (accessed 10 February 2021 using a search strategy for TCIRG1 in the search box) (Warde-Farley et al., 2010) was used to confirm the TCIRG1 gene’s linkage and analyze its connection through other genes in order to anticipate the impact of nsSNPs on specific linked genes. GeneMANIA predicts gene-gene connections using pathways, co-expression, co-localization, genetics, protein interaction, and protein domain similarity.

### 2.3 SIFT and PolyPhen2 Predication

The deleterious/damaging or tolerated nature of isolated nsSNPs will be established first using the SIFT and PolyPhen2 tools. SIFT analyzes protein homology sequences and aligns natural nsSNPs with orthologous and paralogous protein sequences to predict detrimental nsSNPs. If the SIFT score of nonsynonymous SNPs is less than 0.05, they have a deleterious impact on protein function (Ng and Henikoff, 2003). PolyPhen2 assesses a protein’s structural and functional effects by analyzing its sequence and amino acid alterations. When an amino acid is substituted or a mutation in a protein domain is discovered, it divides SNPs into three groups: possibly damaging (probabilistic score >0.15), probably damaging (probabilistic score >0.85), and benign (probabilistic score >0.85). PolyPhen2 can determine the PSIC (position-specific independent count) value of protein variations. If mutants have a direct functional impact on protein function, the diversity in PSIC scores among variations implies that (Adzhubei et al., 2010).

### 2.4 Sequence-Based Prediction and Disease Phenotype Prediction

In-silico tools, PON-P, Mutation Assessor, P-Mut, SNAP2, SNGP-GO, PON-P2, PANTHER, PHD-SNP, SNAP2, PROVEAN, and VarCards algorithms predicted functional implications of the missense mutation as well as confirmatory analysis of the sift and PolyPhen tools. In TCIRG1 protein sequences, to forecast the negative effects of nsSNPS, the PROVEAN algorithm was used. In the case of homologous sequences, a technique like this employs delta alignment scores based on the variant version and a protein sequence comparison. A score of equivalent to or less than 2.5 suggests deleterious nsSNP alignment (Choi et al., 2012). SNAP2 is a neural network-focused classifier. It was used to anticipate how single amino acid alterations in the TCIRG1 protein might affect the protein’s function. This server takes a FASTA sequence and produces a prediction score (range from 100 strong neutral predictions to +100 strong effect prediction) that indicates how likely a mutation is to influence native protein function (Bromberg et al., 2008). PMUT uses neural networks to accurately predict the presence of single amino acid point mutations that cause disease (with an 80 percent success rate in humans). When a FASTA sequence was input into the PMut server, the difference between neutral variants and illness-linked protein sequence was discovered. A score of more than 0.5 indicates that nsSNPs are potentially harmful (Ferrer-Costa et al., 2005). SNP-GO, SNP-PhD (Calabrese et al., 2009) (http://snps.biofold.org/phdsnp/phd-snp.html) are a machine-learning-based approach that uses the conservation scores of multiple sequence alignments to make decisions. The ClinVar dataset was used to create and test the PhD-SNP tool, which typically contains 36,000 harmful and benign SNVs, provides an accuracy index score, and assesses if an SNP effect is deleterious or neutral. PANTHER-PSEP (Tang and Thomas, 2016) (PANTHER -position-specific evolutionary preservation, http://pantherdb.org/apparatuses/csnpScoreForm.jsp) employs a metric that is comparable to, but not identical to, “evolutionary preservation,” in which homologous proteins are employed to retrieve potential ancestral protein sequences at phylogenetic tree nodes. Each amino acid’s roots can be followed to determine how long it has been held in its ancestors in its current state. The PSEP score was categorized into three parts: “probably damaging” (preservation time >450 my), “possibly damaging” (preservation time 200 my), and “probably benign” (preservation time 200 my). VarCARD was used to obtain findings from the MCAP and FATHMM tools. -MKL-coding-pred, LRT, METALR, FATHMM-pred, META SVM, Mutation Assessor, CAAD, DANN, Mutation Taster, META SVM, Mutation Assessor, CAAD, DANN, Mutation Taster. Varcards is a consolidated genetic and medical database that covers human genome coding variants. A number of genomic techniques and databases have been developed to aid in the understanding of genetic variants, notably in nonsynonymous. Varcards, on the other hand, make it easier for scientists, researchers, general practitioners, and geneticists to collect data on a single variant or from a number of different web platforms or databases (Li et al., 2018).

### 2.5 MutPred Predicts Disease-Related Amino Acid Substitutions and Phenotypes

The MutPred internet server (http://mutpred.mutdb.org/) can be used as a search engine to forecast the molecular mechanism of disease caused by amino acid substitutions in mutant proteins. It makes use of a variety of structural, functional, and evolutionary features of proteins. PSI-BLASAT, SIFT, and Pfam profiles, as well as TMHMM, MARCOIL, and DisProt algorithms, were used with three servers. These are some projections for structural damage. The more the scores of all three servers are aggregated, the higher the forecast accuracy (Pejaver et al., 2020).

### 2.6 Structure-based Prediction

I-Mutant 3.0 https://gpcr2.biocomp.unibo.it/cgi/predictors/I-Mutant3.0/I-Mutant3.0.cgi). The ΔΔG Mut dataset from ProTherm was used to pre-train the algorithm. The ΔΔG value (kcal/mol) can be used to determine a single-site mutation that is dependent on a protein structure or sequence. A ΔΔG value less than zero indicates that the variant alters the structure or sequence of a protein. (Capriotti et al., 2005).

### 2.7 Identification of Mutant nsSNPs Position in Different Domains

The InterPro (http://www.ebi.ac.uk/interpro/) tool was used for identification of different conserved domains in the TCIRG1 protein and also mapping of nsSNPs positions in different domains (Hunter et al., 2009). Protein sequence in FASTA format or protein ID was inserted as a query to predict domains and motifs.

### 2.8 Conserved Residues and Sequence Motifs Identification

The human TCIRG1 UniProt protein sequence was BLASTed against the UniprotKB/Swiss-Prot database in NCBI (http://blast.ncbi.nlm.nih.gov/Blast.cgi) and significant alignment was discovered up to 100 sequences. Clustal Omega was used to perform further computational analysis on sequences having more than 50% identity and an E-value of less than 1.00E-20 (Sievers et al., 2011). The amino acid identities were colored using the Clustal color scheme, and Jalview supplied the conservation index at each alignment site (Waterhouse et al., 2009).

### 2.9 ConSurf’s Conservation Predictions for Amino Acids (ConSurf.tau.ac.il)

The evolutionary conservation of amino acids within a protein sequence is calculated using empirical Bayesian inference. Color palettes and conservation scores are included. A score of 1 was given to variable amino acids, while a score of nine was given to the most conserved amino acid. The FASTA sequence of the TCIRG1 protein was submitted for ConSurf analysis (Berezin et al., 2004).

### 2.10 Project HOPE Analysis

Project HOPE is a web server that investigates the structural consequences of the desired mutation. The Hope project provides the changed protein in an observable 3D structure by cooperating with UniProt and DAS prediction algorithms. The protein sequence is used as an input source in Project HOPE, and then a structural comparison with the wild type is performed. Project HOPE is a web server that investigates the structural consequences of the desired mutation. The Hope project provides the changed protein in an observable 3D structure by cooperating with UniProt and DAS prediction algorithms. The protein sequence is used as an input source in Project HOPE, and then a structural comparison with the wild type is performed (Venselaar et al., 2010).

### 2.11 NetSurfP’s Secondary Structure Prediction

Information about amino acid surface and solvent accessibility is needed to determine the interaction interfaces or active sites in a fully folded protein. Binding affinity is affected, and if the protein is an enzyme, catalytic activity is disrupted, when amino acid alterations at such sites are detected (Klausen et al., 2019). NetSurfP-2.0 successfully assesses surface and solvent accessibility, structural disorder, backbone dihedral angles, and secondary structure for amino acid residues. The input is FASTA-formatted protein sequences, and the output is deep neural networks trained on solved protein structures (Klausen et al., 2019). NetSurfP-2.0 is available at http://www.cbs.dtu.dk/services/NetSurfP/.

### 2.12 PTM Sites Prediction

Protein post-translational modifications (PTM) are utilized to predict the protein’s function (Deng et al., 2017). GPSMSP v3.0 (http://msp.biocuckoo.org/online.php) was used to predict methylation sites in the TCIRG1 protein. We used NetPhos 3.177 (https://www.cbs.dtu.dk/services/NetPhos/) (Blom et al., 1999) and GPS 5.078 (https://gps.biocuckoo.cn/) (Xue et al., 2005) to predict possible sites for phosphorylation. The NetPhos 3.1 service predicts Serine, Threonine, and Tyrosine phosphorylation sites in proteins using ensembles of neural networks. Residues in the protein with a score greater than 0.5 indicate phosphorylation. A higher GPS 5.0 score, on the other hand, indicates a higher chance of getting phosphorylated. To estimate probable methylation, ubiquitylation sits, we utilized GPS-MSP 1.0 (Xue et al., 2005) (https://msp.biocuckoo.org/), UbPred (Radivojac et al., 2010) (https://www.ubpred.org), and BDMPUB (https://www.bdmpub.biocuckoo.org). Glycosylation is another important method used by NetOglyc4.0 to predict glycosylation sites (Steentoft et al., 2013). (See http://www.cbs.dtu.dk/services/NetOGlyc/for more information.) Glycosylation sites with a score greater than 0.5 are more likely to be glycosylated.

### 2.13 The FTSite Server (http://FTSite.bu.edu/) Predicts Ligand-Binding Sites

The server FTSite predicted the ligand-binding site in the 3D protein structure. The binding site has been identified in over 94 percent of apoproteins, and the site’s prediction is based on energy. PDB data is used as input for ligand-binding hotspot prediction.

### 2.14 Candidate Variant Filtering

Whole Exome Sequence data of a patient suspected with congenital neutropenia was analyzed for candidate variant filtering and was performed by using BWA, GATK4, and VCF-tools software (Pedersen et al., 2021).

### 2.15 Predicting the Structure of 3D Proteins

Protein modeling is important in the drug development process. Structure prediction from a given sequence with accuracy similar to experimentally resolved structures is the goal of homology modeling (Cavasotto and Phatak, 2009). The inclusion of inserts and loop sequences, which cannot be reliably anticipated in the absence of a three-dimensional (3D) crystal structure, is a limitation of this technique (Ohlson et al., 2004). In the pharmaceutical sector, computational approaches are frequently used to predict 3D protein models (57). To overcome this problem, these methods aid in the prediction of a protein’s tertiary structure based on its amino acid sequence (Katsila et al., 2016). These methods can be classified as either *de novo* or homology modeling, depending on the information available. The most reliable method is template-based modeling, also known as homology modeling or comparative modeling (Cavasotto and Phatak, 2009). Because there were no resolved crystal structures of TCIRG1 available at the time of this research, SWISS-MODEL and HHPred were used to create a homology model for the mutant protein (Schwede et al., 2003; Hildebrand et al., 2009). The 3D structure for the TCIRG1 was also predicated through Phyre2 which is a 3D homology modeling application that predicts 3D models for proteins (http://www.sbg.bio.ic.ac.uk/phyre 2/html/page.cgi?xml:id=index). As 3D models, the wild type and 22 mutants linked to the most harmful nsSNPs were generated (Kelley et al., 2015). Confirmatory molding was conduct of Wild and Mutant TCIRG1 protein through Alpha fold2 which is a highly accurate protein structure predication (Jumper et al., 2021). To compare wild-type TCIRG1 and selected mutations, researchers employed TMalign (https://zhang lab. ccmb.med.umich.edu/TM-align/). Template Modelling score (TMscore), root mean square deviation (RMSD), and structural superposition are all predicted. The TM scores range from 0 to 1, with a higher value indicating more structural similarity. The higher the RMSD values, the greater the difference between mutant and wild-type structures (Carugo and Pongor, 2001). Three mutants with greater RMSD values were submitted to the ITASSER (https://zhang lab.ccmb.med.umich.edu/I-TASSER R/) for further protein 3D structure comparisons (Zhang, 2008; Roy et al., 2010; Yang et al., 2015). Chimera v1.11 to investigate molecular characteristics and interactive visualization of the resulting protein structure (Pettersen et al., 2004). PROCHECK was used to validate the 3D models (Laskowski et al., 1993).

### 2.16 Molecular Dynamic Simulation

For 100 nanoseconds, Desmond, a software from Schrödinger LLC, was used to model molecular dynamics (Bowers et al., 2006; Ferreira et al., 2015). By integrating Newton’s classical equation of motion, MD simulations typically compute atom movements over time. Simulations were used to predict the stability of the protein in the physiological environment. (Hildebrand et al., 2019; Rasheed et al., 2021).

### 2.17 Statistical Analysis

SPSS v23 and MS Excel were used to conduct a correlation study on the predictions made by computational in silico technologies. The significance differences predicted by the various computational techniques were assessed using the Student’s t-test. Significant was defined as a *p*-value of less than 0.01.

## 3 Results

**FIGURE 1:**
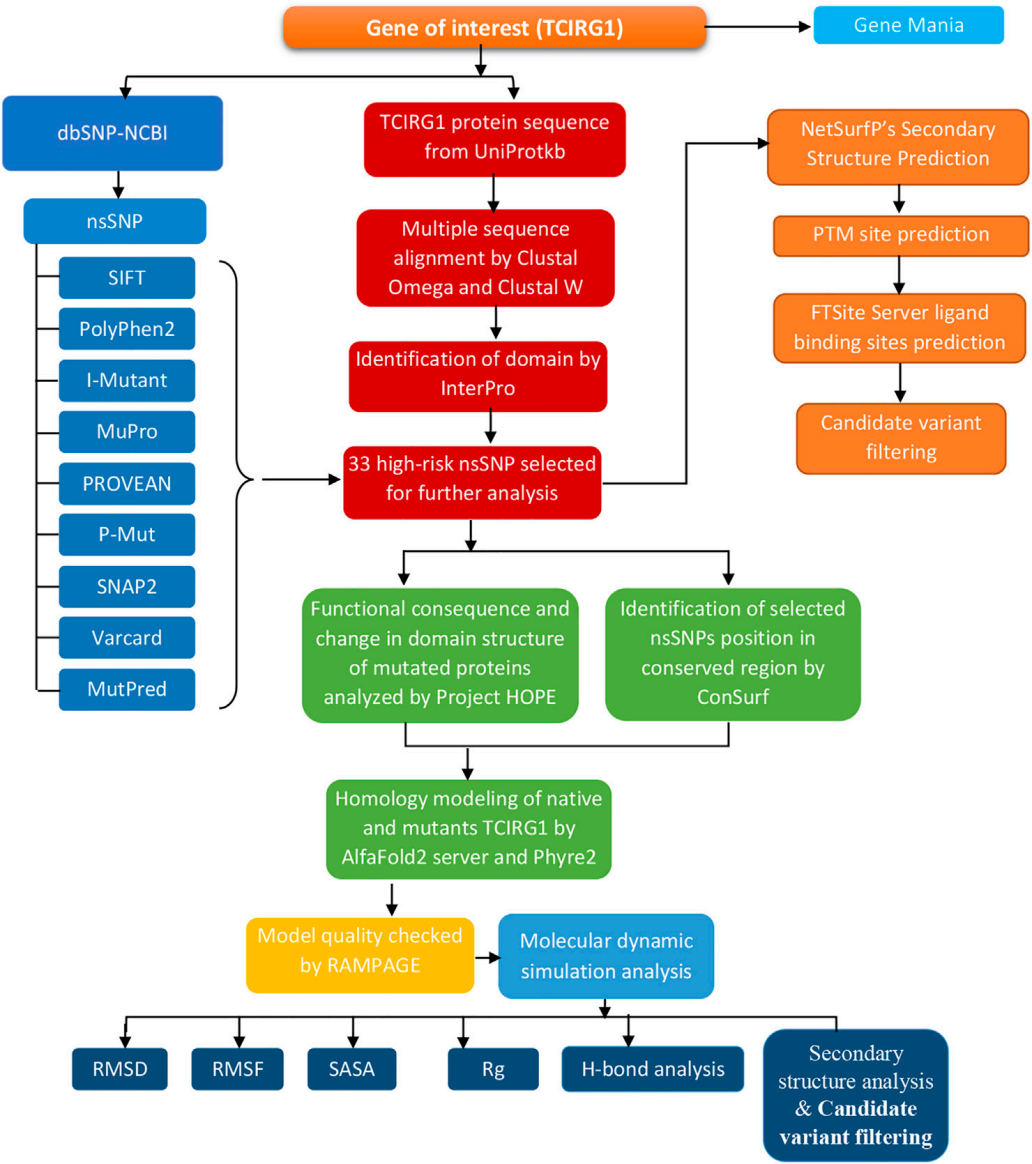
Flowchart for methodology.

The entire approach, tools, and databases used to discover the harmful SNPs in human TCIRG1 and their structural/functional repercussions owing to mutation are summarized in Figure 1.

### 3.1 SNP Annotation

**FIGURE 2:**
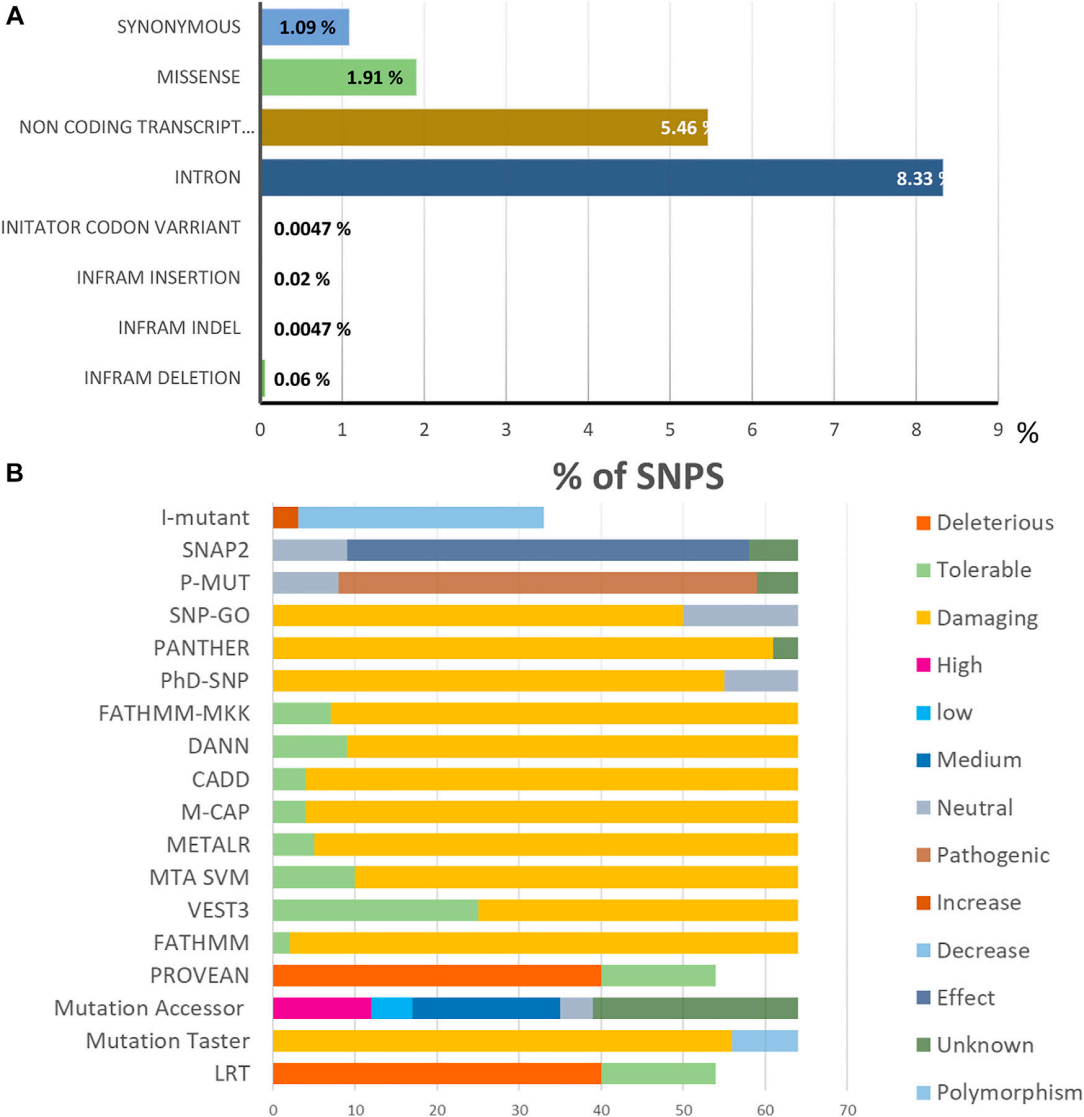
**(A)**. Distribution of SNPs present in the TCIRG1 gene. **(B)**. Prediction results of the 64 SIFT and PolyPhen2 deleterious nsSNPs in the TCIRG1 gene analyzed by the eighteen computational tools.

The NCBI database (http://www.ncbi.nlm.nih.gov/) revealed SNPs in the TCIRG1 gene. It contains 5627 SNPs that were present in Homo sapiens, with 811(1.909%) in coding nonsynonymous regions (missense) and 463 (1.089%) in synonymous sections, as illustrated in Figures 2A,B.

### 3.2 Gene Mania

**FIGURE 3:**
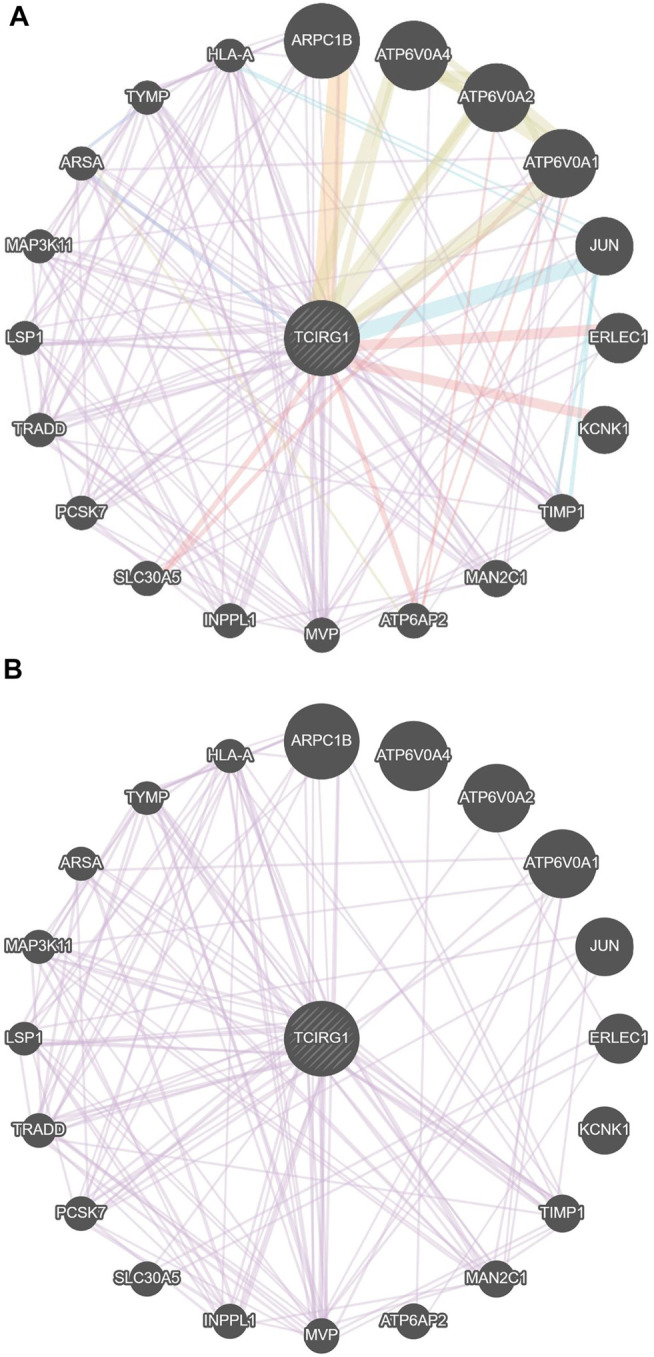
**(A)** Gene–gene interaction of TCGIR1 with other genes proposed by GeneMANIA. **(B)** Co-expression in GenMANIA.

The TCIRG1 gene codes for a protein that can be found in the extracellular matrix of proteins, as well as other compounds. The TCIRG1 protein plays a crucial role in the formation of the lymphatic system. It helps immature lymphangioblasts grow (differentiate) and migrate (migrate), finally forming the lining (epithelium) of lymphatic channels. Our findings revealed that TCIRG1 is co-expressed with 12 genes (MAN2C1, INPPL1, TRADD, ARPC1B, TIMP1, LSP1, TYMP, HLA-A, MVP, ARSA, PCSK7, and MAP3K11) and shared a domain with only three genes (ATP6VOA4, ATP6VO2A, and ATP6VOA1), Physical interaction with seven genes (KCNK1, TRADD, ERLEC1, SLC30AS, ATP6AP2, ATP6VOA2, ATP6VOA1), and co-localization with two genes (ARSA, TYMP) Table 1and Figures 3A,B.

**TABLE 1:** Gene-mania shows the TCIRG1 gene co-expression and shard domain.

Gene symbol	Description	Co-Expression	Shared Domain
MAN2C1	Mannosidase alpha class 2C member 1	Yes	No
INPPL1	Inositol polyphosphate phosphatase like 1	Yes	No
TRADD	TNFRSF1A associated via death domain	Yes	No
ARPC1B	Actin related protein 2/3 complex subunit 1B	Yes	No
TIMP1	TIMP metallopeptidase inhibitor 1	Yes	No
LSP1	Lymphocyte-specific protein 1	Yes	No
TYMP	Thymidine phosphorylase	Yes	No
HLA-A	Major histocompatibility complex, class I, A	Yes	No
MVP	Major vault protein	Yes	No
ARSA	Arylsulfatase A	Yes	No
PCSK7	Proprotein convertase subtilisin/kexin type 7	Yes	NO
MAP3K11	Mitogen-activated protein kinase kinase kinase 11	Yes	No
ATP6V0A4	ATPase H+ transporting V0 subunit a4	No	Yes
ATP6V0A2	ATPase H+ transporting V0 subunit a2	No	Yes
ATP6V0A1	ATPase H+ transporting V0 subunit a1	No	Yes

### 3.3 SIFT and POLYPHEN

A total of 5627 nsSNPs were investigated to see if they influenced protein structure or function in any way. The first step is to figure out which of the nsSNPs is causing the amino acid substitution. SIFT calculates the effect of an nsSNP on protein structure and assesses if the induced amino acid is acceptable at that site. SIFT and PolyPhen predicted 64 nsSNPs that produced amino acid substitutions out of a total of 811 nsSNPs (Table 2 and Supplementary Table S1).

**TABLE 2:** Sift and PolyPhen results of high deleterious nsSNPs in TCIRG1 gene.

ID of nsSNPs	Aa position	SIFT	Score	PolyPhen	Score
rs36027301	R56W	Deleterious	0	Probably damaging	0.999
rs368945298	M546V	Deleterious	0	Probably damaging	0.999
rs115854062	P572L	Deleterious	0	Probably damaging	1
rs150260808	I721N	Deleterious	0	Probably damaging	1
rs137853150	G405R	Deleterious	0	Probably damaging	1
rs137853151	R444L	Deleterious	0	Probably damaging	1
rs147580611	F610S	Deleterious	0	Probably damaging	1.00
rs148921764	E722K	Deleterious	0	Probably damaging	1.00
rs140963213	A417T	Deleterious	0.002	Probably damaging	1
rs144775787	A778V	Deleterious	0.46	Probably damaging	0.883
rs145080707	R213W	Deleterious Low	0.012	Probably damaging	1
rs150648332	R57H	Deleterious	0.001	Probably damaging	1.00
rs150260808	I721N	Deleterious	0	Probably damaging	1
rs201329219	R109W	Deleterious	0.014	Probably damaging	1.00
rs367703865	R191H	Deleterious	0.32	Probably damaging	0.999
rs371214361	S532C	Deleterious	0.001	Probably damaging	1.00
rs199914625	S474W	Deleterious	0	Probably damaging	1
rs200851583	G458S	Deleterious	0	Probably damaging	1
rs371658110	G192S	Deleterious	0.003	Probably damaging	1.00
rs370319355	R50C	Deleterious	0	Probably damaging	1
rs376351835	F529L	Deleterious	0.013	Probably damaging	1.00
rs371004297	G379S	Deleterious	0.011	Probably damaging	1.00
rs200209146	N730S	Deleterious	0.022	Probably damaging	1.00
rs200415611	V375M	Deleterious	0.001	Probably damaging	1.00

rs367818260	T314M	Deleterious	0.001	Probably Damaging	1.00
rs375809635	R363C	Deleterious	0	Probably damaging	1.00
rs138305091	A732T	Deleterious	0.001	Probably damaging	1.00
rs138308753	F51S	Deleterious	0	Probably damaging	0.996
rs141095902	A717D	Deleterious	0.002	Probably damaging	0.963
rs369264588	D517N	Deleterious	0	Probably damaging	1.00
rs371907380	R92W	Deleterious	0	Probably damaging	1.00
rs373988992	T368M	Deleterious	0	Probably damaging	1.00
rs142606750	R757C	Deleterious	0.003	Probably damaging	1.00
rs367818260	T314M	Deleterious	0.001	Probably damaging	1.00
rs118141250	V52L	Deleterious	0.11	Probably damaging	0.924

Threshold: Sift: < 0.05 Polyphen2: >0.8 (PSIC >0.5) or Benign (PSIC <0.5).

### 3.4 The Most Deleterious SNPs Identified in TCIRG1

#### 3.4.1 Functional SNPs in Coding Areas Were Identified

The various computational prediction tools that were used in this study, are illustrated in Figure 2B to identify significant nsSNPs in TCIRG1. The nsSNPs in table 3 are variations that are predicted to be deleterious by all algorithms. FATHMM-MKL. While they are regarded as high-risk pathogenic nsSNPs, SNP-GO, PHD-SNP, PANTHER, SNAP2, P-MUT PROVEAN, FATHMM, LRT, M-CAP, CAAD, META SVM, METALR, Mutation Assessor, and Mutation Taster are considered high-risk pathogenic nsSNPs. There are a variable number of deleterious SNPs in each technique. SIFT classed 118 and PolyPhen 64 nsSNPs as harmful or deleterious, although PolyPhen did not show any of the 58 nsSNPs that were deleterious. Sift classified deleterious with a threshold of >0.5, and both SIFT and Polyphen confirmed 34 as deleterious. In a total of 118 unique predicted nsSNPs in the TCIRG1 gene, VEST three indicated the fewest six nsSNPs (10%) as destructive or detrimental, and 51 as tolerated. PolyPhn, FATHMM, M-CAP, and PANTHER had the largest percentage of harmful predictions. Using the SNAP2 technique, 41 were found to be harmful (71%) and 16 were found to have no effect (SNAP2 score of 100). The deleterious and damaging effects of 54 (92%) nsSNPs on TCIRG1 protein were predicted using the PANTHER program, with 48 nsSNPs being probably damaging, six nsSNPs being possibly damaging, and three nsSNPs being probably benign (time >450my possibly damaging” (450my > time >200my, “probably benign” (time 200my). PROVEAN is a program that predicts the impact of SNPs on a protein’s biological function. 22 (38 percent) nsSNPs in the TCIRG1 gene were projected to be severely detrimental, while 35 nsSNPs were neutral, according to PROVEAN’s criterion (>-2.667). With a threshold of (>0.65 (5.545 to 5.975 (higher score > more damaging), the Mutation Assessor classified 24 nsSNPs as deleterious, with 12 high, 17 medium, five low, and 19 as no findings. FATHNMM and FATHMM-MKK (<0.5), CADD (>15) DANN (>0.5), Mutation Taster (<0.5), and with respective scores show all above than (75–90%) nsSNPs as deleterious/damaging. while P-Mut predicated 45 (75.21%) deleterious, 07 neutral, and 5 with no result with a cut off (<0.5). LRT predicted 42 (77%) deleterious nsSNPs with a score (>0.001) and 13 as Neutral. PhD-SNP, SNP-GO, and M-CAP identified 47 (82%), 35 (61%), and 54 (94.73%) as deleterious, respectively. MetalR and MTA-SVM identified 10 (17%) and 37 (64%) nsSNPs as deleterious. Based on the substitution position-specific scores using PANTHER, PROVEAN score, SIFT score, SNPs&GO, FATHMM, LRT, M-CAP, VEST3, CAAD, METALR, Mutation Assessor, Mutation Taster, FATHMM-MKL, PHD-SNP score and PolyPhen server, PSIC score (>0.5). A group of 15 nsSNPs P572L, M546V, I721N, F610S, A732T, F51S, A717D, E722K, R57H, R109W, R191H, S532C, G192S, F529L, H804Q were all considered highly deleterious by all state-of-the-art methods. While only LRT disagrees with the result of A717D by other tools. All of the prediction algorithms’ findings were found to be statistically significant and strongly correlated. The *p*-value for the Student *t*-test between the tools was 0.001. Results of prediction tools and their significance are shown in (Supplementary Table S2).

**TABLE 3:** TMscore and RMSD values of 56 deleterious nsSNPs in TCIRG1.

SNP-ID	Residual Change	TM-score	RMSD Values	SNP-ID	Residual Change	TM-score	RMSD Values
rs199902030	P572L	0.99626	0.78	rs121908252	R56W	0.99621	0.78
rs200149541	M546V	0.99626	0.78	rs121908254	G379C	0.99435	0.58
rs372499913	I721N	0.99760	0.53	rs147974432	R757C	0.99790	0.48
rs267605221	F610S	0.99312	0.81	rs192224843	N730S	0.99275	0.84
rs374941368	A732T	0.99621	0.78	rs115982879	V375M	0.99743	0.54
rs375717418	F51S	0.99626	0.78	rs139059968	T314M	0.99626	0.78
rs80008675	A717D	0.99661	0.73	rs141125426	D517N	0.99785	0.49
rs149792489	E722K	0.99830	0.46	rs147208835	R92W	0.96213	0.89
rs116675104	R57H	0.99790	0.48	rs147681552	T368M	0.99626	0.78
rs121908250	R109W	0.99626	0.78	rs148498685	A417T	0.99790	0.48
rs121908251	R191H	0.99785	0.49	rs149531418	R363C	0.99626	0.78
rs121908251	S532C	0.99092	0.81	rs149531418	A778V	0.99661	0.76
rs149792489	G192C	0.99626	0.78	rs147208835	R50C	0.99621	0.78
rs116675104	F529L	0.99435	0.58	rs121908250	H804Q	0.99790	0.48
rs121908251	G405R	0.99674	0.62	rs149792489	S474W	0.99760	0.53
rs116675104	G458S	0.99674	0.48	rs121908250	R444L	0.99270	0.84
rs121908251	R56P	0.99657	0.48				

### 3.5 MutPred2 Predicts Pathogenic Amino Acid Substitutions

MutPred2 assesses a variety of molecular characteristics of amino acid residues in humans to identify whether a substitution is disease-related or not. It assigns a score based on the chance that a change in amino acid will affect the protein’s function. A MutPred2 score of 0.8 or higher is considered highly confidential, while the pathogenicity prediction cutoff is 0.5. The prediction score for all of the substitutions was less than 0.5. The MutPred2 results are available in (Supplementary Table S6).

### 3.6 I-Mutant 3.0 Predicts the Stability of the Mutated Protein due to SNPs

The effects of TCGIR1 high-risk nsSNPs on protein stability and function were predicted using the web program I-Mutant 3.0 (Supplementary Table S3) The results showed that (G405R, S474W, and A778V) have increased stability while (P572L, M546V, I730N, F610S, A732T, F51S, A717D, E722K, R57H, R109W, R191W, S532C, G192S, F529L, H804Q, G458S, R444L, R56P, G379S, R757C, N730S, V375M, T314M, D517N, R92W, T368M, A417T, R363C, R56W, and R50C) showed decreased stability.

### 3.7 Identification of Domains in TCIRG1

InterPro tool was used to locate domain regions in TCIRG1 and to identify the location of nsSNPs in different domains. This tool provides a functional analysis of proteins by classifying them into families. It also predicts the presence of domains and active sites. It has been reported a three domain: such as the V-TYPE PROTON ATPASE 116 KDA SUBUNIT A ISOFORM 3 (1–828), cytoplasmic and non-cytoplasmic are found in TCIRG1. The 33 nsSNPs and fifteen highly deleterious that we have selected are located in V-TYPE PROTON ATPASE and cytoplasmic domains.

### 3.8 SNPs in TCIRG1 Protein Are Linked to Highly Conserved Buried (Structural) and Exposed (Functional) Amino Acid Residues

**FIGURE 4:**
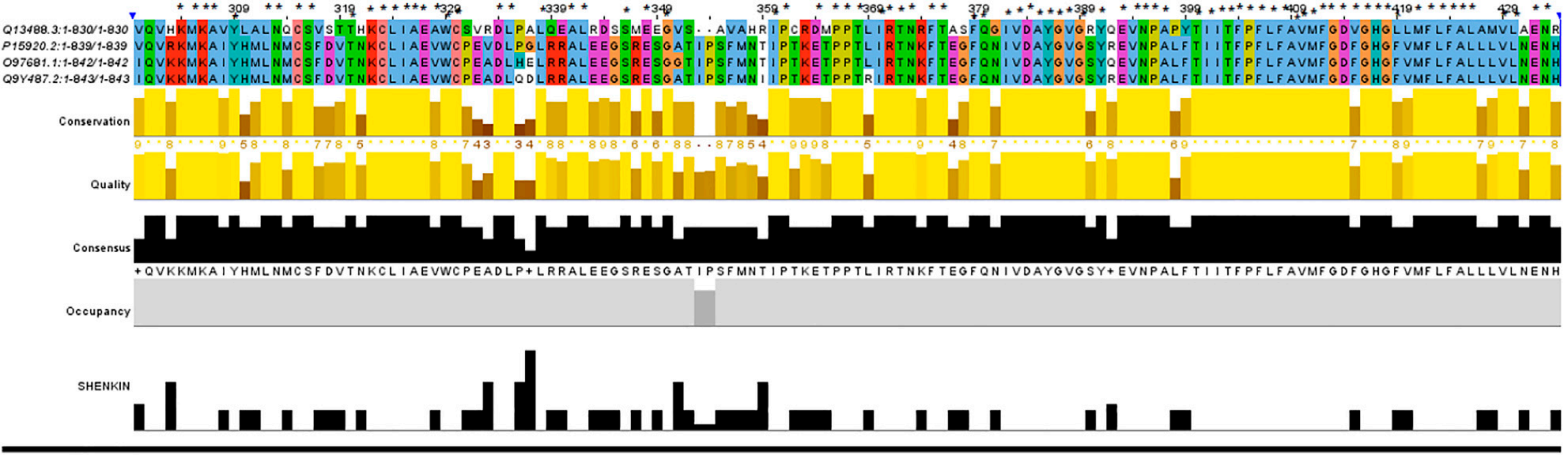
In ABWGB and Q3MI99, amino acid alignment of human TCIRG1 (UniProt ID: Q6UXH8) and homologs in phylogenetically adjacent species. Residues with an asterisk (*) mark indicate evolutionarily conserved amino acids, while solid horizontal bars indicate conserved sequence patterns. The conservation index at each alignment point was provided by Jalview, and the amino acid identities were colored according to the Clustal color scheme.

TCIRG1 (ATPase H + Transporting V0 Subunit A3, T Cell Immune Regulator 1) is a protein-coding gene that codes for ATPase H + Transporting V0 Subunit A3. Autosomal Recessive 1 and Autosomal Recessive Malignant Osteopetrosis TCIRG1 is associated with disorders like osteopetrosis. The lysosome cycle and the synaptic vesicle cycle are two related pathways. This gene, which is located on chromosome 11, is 830 amino acids long and has a molecular mass of 92968 Da. TCIRG1 sequence-based structural-functional investigation was analyzed using Clustal Omega-based multiple sequence alignment analysis. The Uniprot Knowledgebase was used to retrieve the TCIRG1 protein sequence (Uniprot ID: Q13488). After being BLASTed against UniprotKB/SwissProt entries, the TCIRG1 protein sequence was aligned using Clustal Omega with default settings. This gene, which is located on chromosome 11, is 830 amino acids long and has a molecular mass of 92968 Da. TCIRG1 sequence-based structural-functional investigation was analyzed using Clustal Omega-based multiple sequence alignment analysis. The Uniprot Knowledgebase was used to retrieve the TCIRG1 protein sequence (Uniprot ID: Q13488). After being BLASTed against UniprotKB/SwissProt entries, the TCIRG1 protein sequence was aligned using Clustal Omega with default settings. The highly conserved amino acid residues in human TCIRG1 protein were K304, M305, K306, A307, Y309, L312, N313, C315, S316, T320, K322, K322, C323, L324, I325, A326, E327, W329, C330, D334, L335, L338, A341, L342, S346, E348, S350, I360, P361, P366, P367, T368, I369, R371, T372, N373, F375, F379, Q380, I382, V383, D384, A385, Y386, G387, V388, G389, Y391, E393, V394, N395, P396, A397, T400, I401, I402, I403, F404, P405, F406, L407, F408, A409, V410, M411, F412, G413, D414, G416, H417, G418, L419, M421, F422, L423, F424, A425, L426, V429, L430, and E432. There are eighty-one different conserved residues Results can be seen in Figure 4.

### 3.9 Conservation Analysis

**FIGURE 5:**
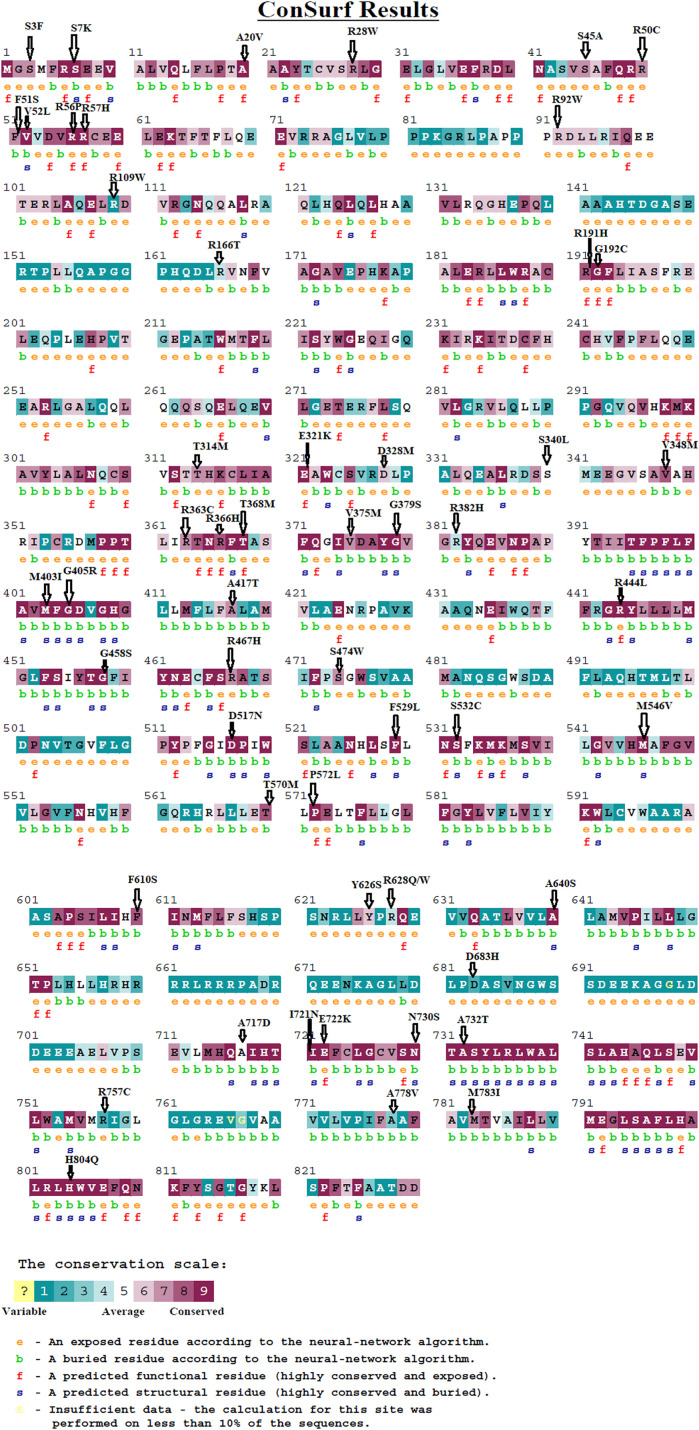
The evolutionary conservation of amino acids in the TCIRG1 gene was assessed using the ConSurf service. A value of 1 indicates a high variability region. The value grows as the region becomes more conserved until it reaches 9.

We used the ConSurf web server to look at the conservation of TCIRG1 residues. According to the results of the ConSurf investigation, 22 deleterious missense SNPs are found in highly conserved areas (7-8–9). The other 16 (S7K, V52L, G379S, M403I, G405R, G458S, D517N, F529L, S532C, M546V, A640S, D683H, I732N, N730S, A732T, and H804Q) were predicted as functional and exposed residues, while the other 10 (A20V, R56P, R57H, R191H, G192C, E321K, R366H, T368M, R444L, and E722K) were predicted as functional and exposed residues and the other 16 (S7K, V52L, G379S, M403I, G405R, G458S, D517N, F529L, S532C, M546V, A640S, D683H, I732N, N730S, A732T, and H804Q) were predicted as buried and structural residues. The 18 (S3F, R28W, S45A, R50C, R92W, R109W, R166T, T314M, D328M, S340L, R363C, R382H, R467H, S474W, P572L, Y626S, R628W, and R757C) were predicted as exposed and the other 9 (F51S, V348M, V375M, A417T, T570M, F610S, A717D, A778V, and M783I) were buried residues. The results are shown in Figure 5.

### 3.10 Project Hope

All of the predicating techniques projected negative consequences for 15 high-risk pathogenic TCIRG1 nsSNPs, hence HOPE was utilized to forecast their effects. The hop was based on the size, spatial, charge, hydrophobicity, structure, and function of amino acids. Seven mutant amino acids were smaller than their wild-type counterparts, while eight were larger. The charge was switched from positive to neutral at three different locations. Six alterations exhibited an increase in hydrophobicity, while the other did not. This finding implies that amino acid changes at these locations modify protein structure and interactions with other molecules, influencing protein function. The outcomes can be seen in the graph below (Supplementary Table S8).

### 3.11 TCIRG1 Secondary Structure and Surface & Solvent Accessibility of Residues Analysis by NetSurfP-2.0

**FIGURE 6:**
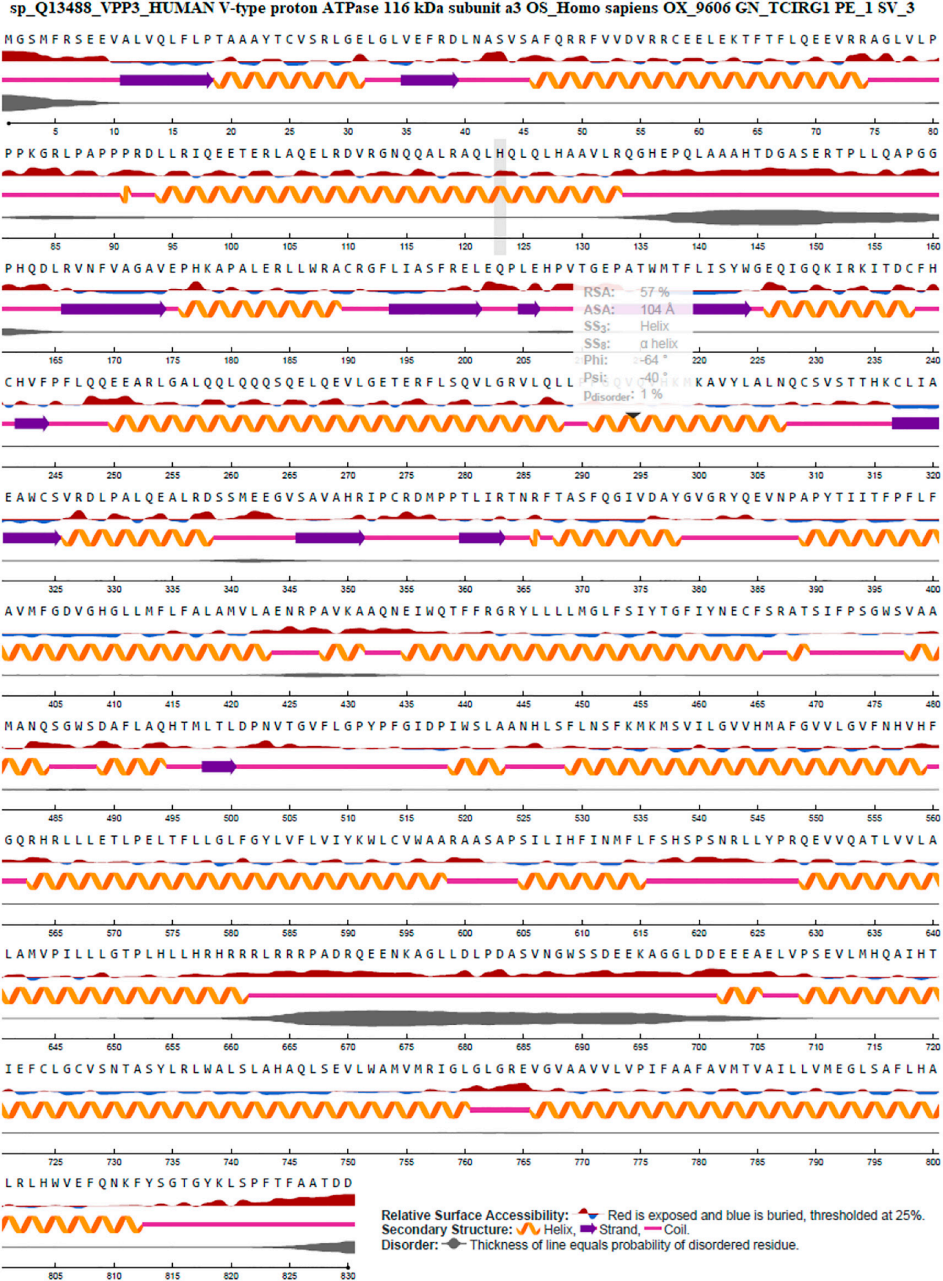
Secondary structure prediction by Net-SurfP-2.0.

The surface accessibility (exposed or buried) of amino acids in a given protein was predicted using NetSurfP-2.0, which determines the relative and absolute accessible surface area of each residue. It can also predict protein secondary structure. Relative Surface Accessibility: With a threshold of 25%, red upward elevation implies residue exposure, whereas sky blue denotes buried residue. A helix is represented by an orange spiral, a strand is represented by an indigo arrow, and a coil is represented by a pink straight line. The disorder is represented as a bloated black line, with the thickness of the line equaling the probability of disordered residue. Figure 6: NetSurfP-2.0 results.

### 3.12 PTMs (Posttranslational Modifications) Predictions

This was done with GPSMSP 3.0, which predicted that no sites in TCIRG1 were methylated. TCIRG1 phosphorylation sites predicted by GPS 3.0 and NetPhos 3.1 are included in Supplementary Table S1. NetPhos 3.1 projected phosphorylation potential for 62 residues (Ser23, Thr: 22, Tyr: 17). GPS 3.0, on the other hand, suggested that 18 residues (Ser: 12, Thr: 06, Tyr: 00) may be phosphorylated. For ubiquitylation prediction, BDMPUB and UbPred were utilized. UbPred projected that none of the lysine residues would be ubiquitinated, but BDMPUB predicted that none of the lysine residues would be ubiquitinated. None of the BDMPUB predictions were found in a highly conserved or detrimental nsSNP region. Table 2, Supplementary Table S2 shows the results achieved. Potential glycosylation sites were predicted using NetOGlyc4.0. Positions 43, 145, 152, 346, and 474 in wild-type TCIRG1 protein were predicted to be glycosylated with scores of 0.513,032, 0.554,065, 0.884,332, 0.830,233, 0.585,103, and 0.511,937. Interestingly, mutant S532C lost its glycosylation site at position 532, but mutant N730S gained it at position 730. Supplementary Table S5 contains all of the scores for the wild type and mutants.

### 3.13 FTSite Predicts Ligand-Binding Sites

The ligand binding sites were predicted using FTSite algorithms, which were then visualized and analyzed using Pymol. Using this technique, three ligand-binding sites in human TCRIG1 protein were found (Supplementary Figure S11). Site 1 has 14 residues, while sites two and three each had 9, 13, and so on. In the fifty-six replaced positions, none of the substitutions in the SIFT server’s expected ligand-binding sites are detected (Supplementary Table S7). In that sequence, the expected binding sites are colored pink, green, and purple. Residues within 5 nm of the binding site are represented using a ball and stick representations of side-chain atoms. The atoms are colored according to their elements, with carbon matching the binding site’s color. RaptorX Binding ligand-binding site prediction servers were used to predict ligand-binding sites in the TCIRG1 protein. A pocket multiplicity value of greater than 40, according to the RaptorX Binding server, indicates a precise prediction. The TCIRG1 protein has the maximum pocket multiplicity of 20, with an expected CVM (2+) cation ligand connected to residues L801 H804 W805 D822 D830.

### 3.14 3D Modeling of TCIRG1 and Its Mutants

**FIGURE 7:**
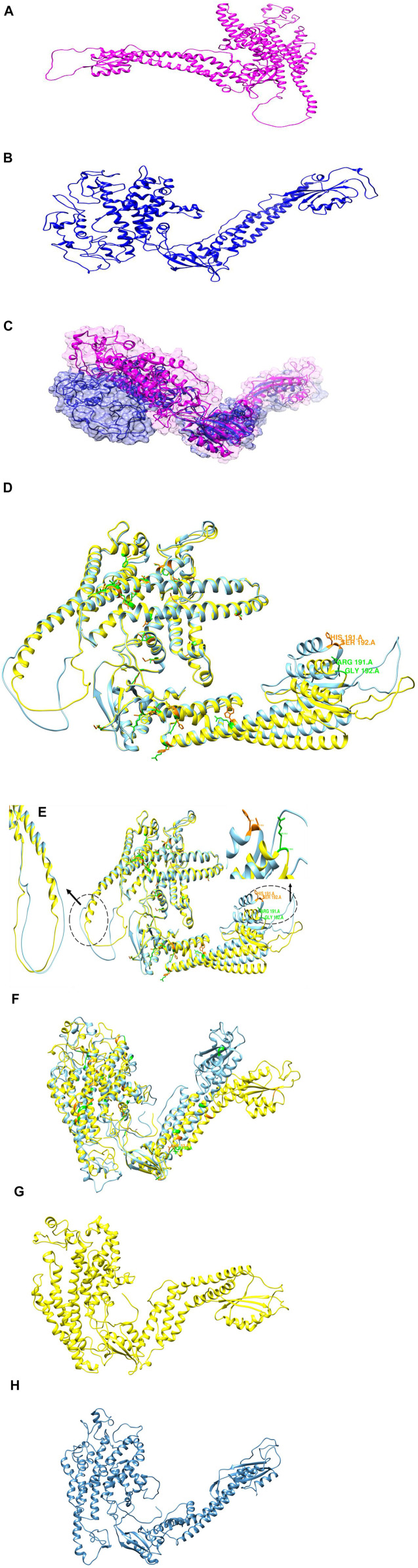
**(A)** 3D structure of wild type protein predicted by AlphaFold2. **(B)** 3D predicted structure of Mutant protein. **(C)** superimposition of 3D structure of Mutant (blue) and Wild Type Magenta. **(D)** Superimposition of initial 3D structure of Mutant (cyan) and Wild Type (yellow). **(E)** superimposition of 3D structure of Mutant (cyan) and Wild Type (yellow). **(F)** Superimposition of 3D structure of Mutant (cyan) and Wild Type (yellow) at 50 ns. **(G)** 3D structure of Wild type at 100 ns. **(H)** 3D structure of Mutant at 100 ns.

The protein 3D model was predicted by HHpred, Phyre2 and AlphaFold2 while the wild-type structure was predicted by AlphaFold2 available in uniport with Q13488 ID. The mutant structures predicted by HHpred and proceed with MD Simulation and similarly, the structure of mutant was also predicted by Alphafold2 and also proceed for 100ns MD simulation for further analysis and validation (Figure 7A-H. These structures proceeded with MD simulation for further analysis and validation. Phyre2 was also used to generate 3D structures of the wild-type TCIRG1 protein as well as 56 mutations. nsSNP replacements in the TCIRG1 protein sequence were made separately and then submitted to Phyre2, which predicted the mutant proteins’ 3D structures. C6VQ7A was chosen as a template for 3D model prediction by Phyre2 because it was the template with the highest similarity, according to the Phyre2 server. For each mutant model, TM scores and RMSD values were determined. The TM-score measures topological similarity, whereas the RMSD values measure the average distance between the carbon backbones of natural and mutant models. Higher RMSD values indicate that the mutant structure differs from that of the wild type. The mutant R92W (rs371907380) has the highest RMSD value of 0.89B, followed by R444L (rs137853151), N730S (rs200209146), and S532C (rs371214361) with 0.84B, 0.84B, and 0.81B, respectively. F610S, M546V, and P572L have RMSD values of 0.B, 0.78B, and 0.78B, respectively, indicating no structural differences from wild type. Other nsSNPs showed slight variation which included I721N (0.53B RMSD), A732T (0.78B RMSD), R51C (0.78B RMSD), A717D (0.73B RMSD), E722K (0.46B RMSD), R57H (0.48B RMSD), R109W (0.78B RMSD), R191H (0.49B RMSD), G192C (0.78B RMSD), F529L (0.58B RMSD), H804Q (0.48B RMSD), G405R (0.48B RMSD) S474W (0.53B RMSD), G458S (0.48B RMSD), R56P (0.48B RMSD), R56W (0.78B RMSD), G379C (0.58B RMSD), R757C (0.48B RMSD), V375M (0.54B RMSD), T314M (0.78B RMSD), D517N (0.49B RMSD), T368M (0.78B RMSD), A417T (0.40B RMSD), R363C (0.78B RMSD), A778V (0.76B RMSD) and R50C (0.78B RMSD). Table 3 shows the TMscores and RMSD values. The four nsSNPs with the greatest RMSD values (R92W, R444L, N730S, and S532C) were chosen and submitted to ITASSER for remodeling. The protein structure produced by the ITASSER is the most dependable since it is the most powerful modeling tool by using Chimera 1.11. Phyre2 Wild type mutant and three mutations superimposed on the wild-type TCIRG1 protein are shown in Supplementary Figure S9 while validation results for the wild and mutant versions of the 3D models were good, and the Ramachandran plots may be found in the (Supplementary Figure S10).

### 3.15 Clinical Identification of Deleterious V52L nsSNP in a Patients Having Symptoms Related to PID

One of our patient who was a Russian kid 7 years old was suspected for Congenital *Neutropenia*, having symptoms related to chronic infections (right-side catarrhal otitis, acute rhinitis, and chronic tonsillopharyngitis). Whole genome sequencing (WGS) was conducted and the result showed no mutations for the suspected disorder. Analysis of the whole genome sequencing data of the patient was carried out using the BWA, GATK4, VCFtools software. An analysis of the so-called “candidate variant filtering” was performed using the ANNOVAR software and the Combined Annotation Dependent Depletion (CADD) database, and its results are schematically presented in Figure 28. The first filtration step was to remove all synonymous SNV, non-frames InDels and embodiments are marked as “NA” or “unknown”. A total of 270 were identified variants or INDEL SNV. Then, the identified variants were filtered by overlaying on the known 351 PID genes and known congenital neutropenia genes. Selected 111 variants were retained to search for more possible ones. After eliminating the common variants, whose Minor allele frequency (MAF)>0.01 for The Exome Aggregation Consortium (ExAC), 1000g and The Genome Aggregation Database (gnomAD), a total of six rare variants remained. To select pathogenic mutations, CADD, the Functional Analysis through Hidden Markov Models (FATHMM), and Protein Variation Effect Analyzer (PROVEAN) models were used, and finally, four mutations that are likely to lead to the development of the disease in this patient were predicted. In particular, a mutation (g. 68041789G > C) was identified in the TCIRG1 gene. The mutation was V52L which in our Insilco analysis this mutation was predicted through many algorithm tools and this mutation was found to disturb the function and structure of TCIRG1 protein.

### 3.16 Simulation

The wild type and mutant proteins were preprocessed using Protein Preparation Wizard of Maestro, which included complex optimization and minimization. All the systems were prepared using the System Builder tool. TIP3P, a solvent model with an orthorhombic box, was chosen. (Transferable Intermolecular Interaction Potential three Points). In the simulation, the OPLS 2005 force field was used (Rasheed et al., 2021). To make the models neutral, counter ions were introduced. To mimic physiological conditions, 0.15 M sodium chloride (NaCl) was added. The NPT ensemble with 300 K temperature and 1 atm pressure was chosen for the entire simulation. The models were relaxed before the simulation. The trajectories were saved for examination after every 100 ps, and the simulation’s stability was verified by comparing the protein and ligand’s root mean square deviation (RMSD) over time.

**FIGURE 8:**
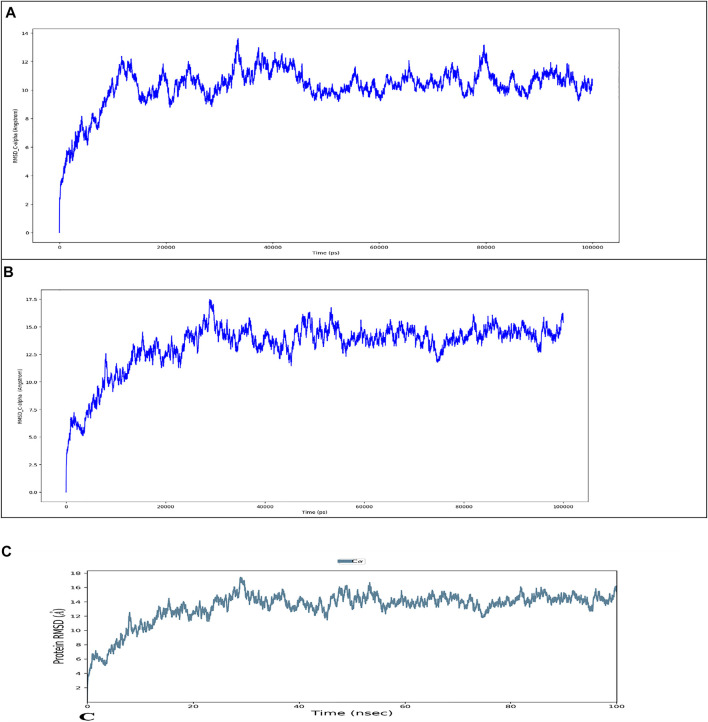
Root mean square deviation (RMSD) of the C-alpha atoms of Wild Type **(A)** and Mutant by HHpred **(B)** and Mutant by Alphafold2 **(C)** with time. The left *Y*-axis shows the variation of proteins RMSD through time.

**FIGURE 9:**
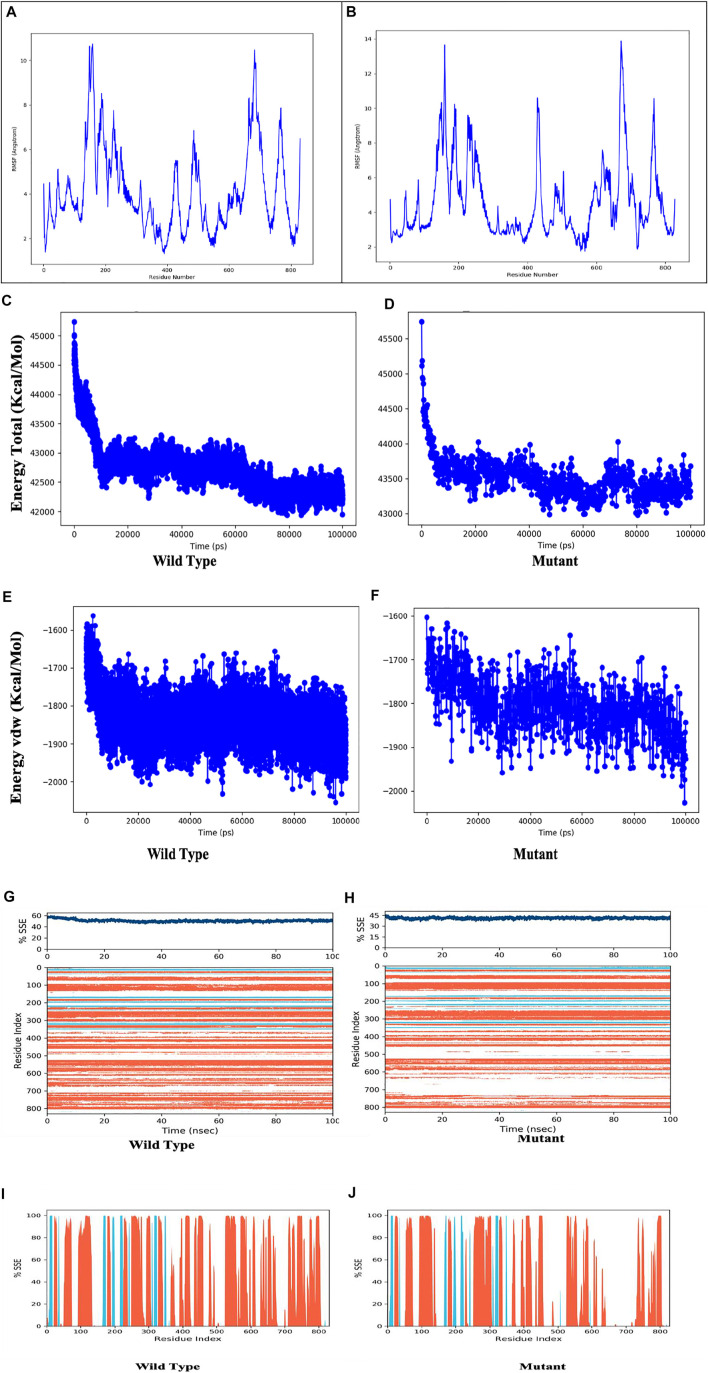
Residue wise Root Mean Square Fluctuation (RMSF) of **(A)** Wild Type protein, **(B)** Mutant protein, total energy of the wild type**(C)** compared to mutant protein (D, Vander Waal’s energy of the wild type**(E)** compared to mutant protein **(F)**, Secondary structure element (SSE) percentage of the wild type **(G)** and mutant protein **(H)**, Distribution of SSE by residue index across the protein structures, alpha helices (orange), beta strands (cyan) and loops (white) along the simulated time of 100 ns.

Figure 8 depicts the evolution of RMSD values for the C-alpha atoms of protein over time. The plot shows that the protein reaches stability at 20,000 ps. After that, for the length of the simulation, fluctuations in RMSD values for wild type remain within 2.0 Angstrom, which is acceptable (Pedersen et al., 2021). The mutant protein RMSD values fluctuate within 3.5 Angstrom after they have been equilibrated. These findings indicate that the mutant protein has higher RMSD throughout the simulation period. On the RMSF graphic (Figure 9A, B), peaks represent portions of the proteins that fluctuate the most during the simulation. Protein tails (both N- and C-terminal) typically change more than any other part of the protein. Alpha helices and beta strands, for example, are usually stiffer than the unstructured section of the protein and fluctuate less than loop portions. According to MD trajectories, the residues with greater peaks belong to loop areas or N and C-terminal zones. Alpha-helices and beta-strands are monitored as secondary structure elements during the simulation (SSE). The graph above depicts the distribution of SSE by residue index across the protein structures. The mutant and wild total energy, Vander Waal’s energy, and Secondary structure element (SSE are shown in Figure 9C-J as mutant show different total energy and Vander Waal’s energy from the wild.

### 3.17 Intramolecular H-Bonds can Be Detected Throughout the Simulation

**FIGURE 10:**
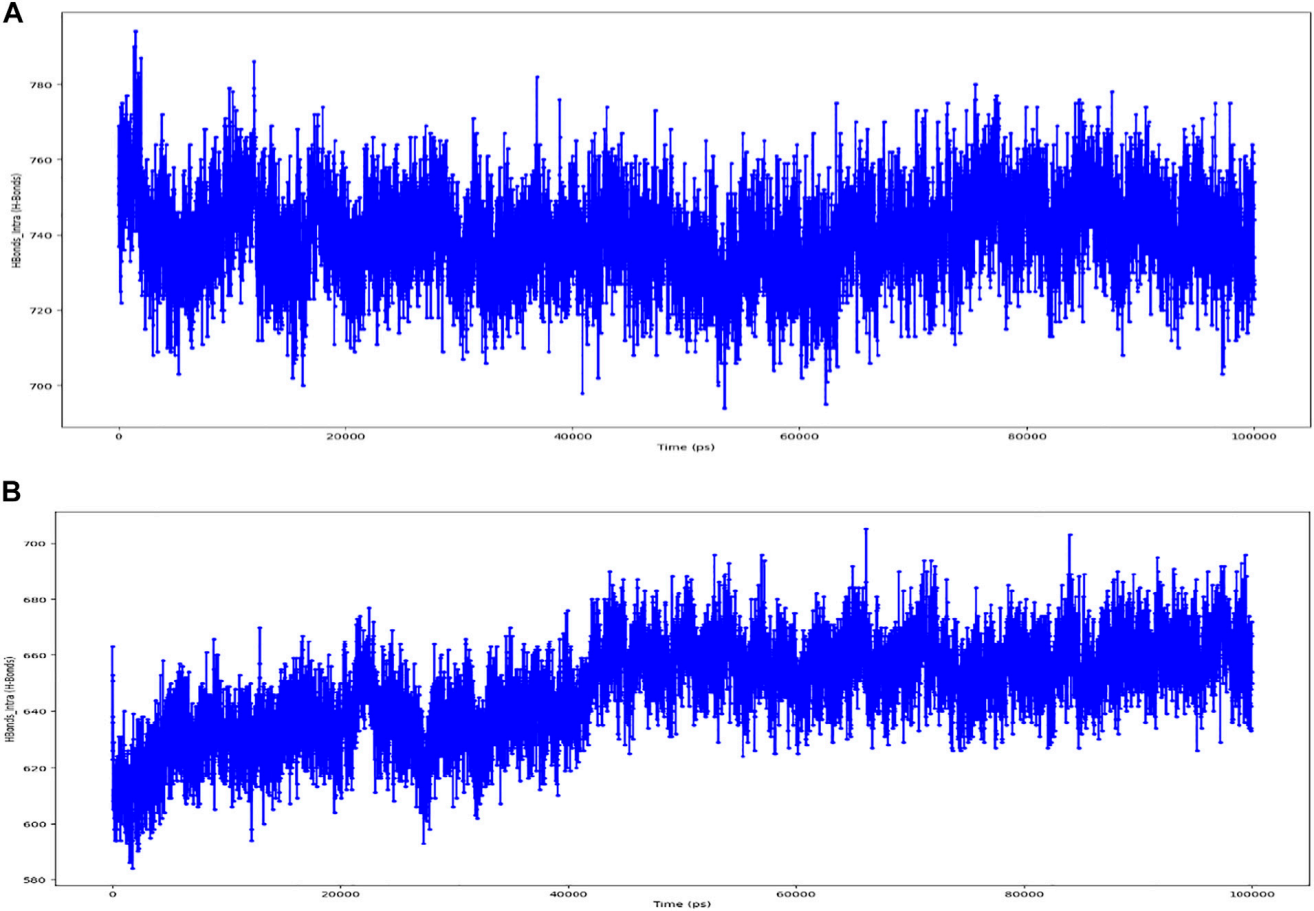
**(A)** timeline representation of the interactions and contacts (H-bonds) Wild Type. **(B)** timeline representation of the interactions and contacts (H-bonds) of Mutant.

**FIGURE 11:**
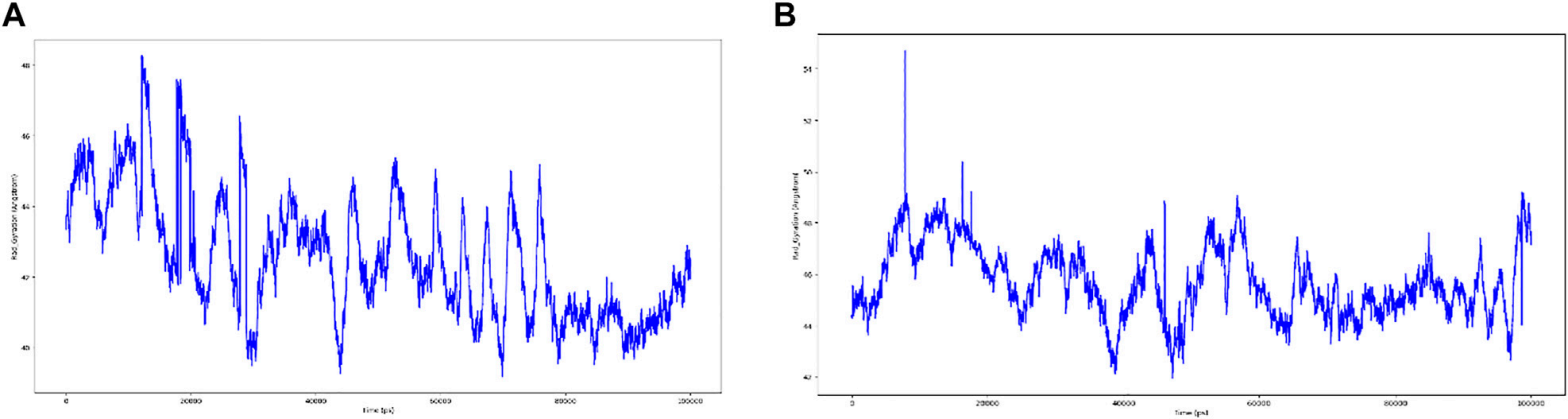
Radius of gyration, **(A)** Wild Type Protein, **(B)** Mutant Protein.

As seen in Figure 10, most of the significant intramolecular interactions discovered by MD are hydrogen bonds. A timeline depicts the interactions and contacts. The distribution of atoms in a protein around its axis is known as the radius of gyration (Rg). Rg is the length that reflects the distance between the rotating point and the place where the energy transfer has the greatest effect. This conceptual idea also aids in the identification of diverse polymer kinds, such as proteins. The two most important markers for forecasting the structural activity of a macromolecule are the calculation of Rg and distance calculations. The pace of folding of a protein is directly related to its compactness, which may be tracked using an advanced computer approach for determining the radius of gyration Figure 11.

## 4 Discussion

A number of studies have found a relationship between SNPs in the TCIRG1 gene and osteopetrosis and congenital neutropenia. (Sobacchi et al., 2001; Susani et al., 2004; Makaryan et al., 2014; Scimeca et al., 2003; Rosenthal et al., 2016). TCIRG1 still has far too many SNPs that could play an impact on the disorders caused by this gene. We looked at TCIRG1’s nsSNPs to discover which ones were the most detrimental and could be linked to Osteopetrosis, congenital neutropenia, and other immune-related diseases in this study. In this work, the dbSNP database revealed 811 nsSNPs in the TCIRG1 gene. Sixty-four nsSNPs in the TCIRG1 gene were validated as high-risk detrimental by SIFT and PolyPhen. The top fifteen high-risk nsSNPs in (Table 4) have been verified as extremely harmful by all state-of-the-art prediction techniques employed in the study. These fifteen nsSNPs (P572L, M546V, I721N, F610S, A732T, F51S, A717D, E722K, R57H, R109W, R191H, S532C, G192S, F529L, and H804Q) have not yet been connected to TCIRG1 gene-related osteopetrosis and congenital neutropenia, however, they could be utilized as a markers nsSNPs variants whenever diagnosing disorders related with TCIRG1 gene. These nsSNPs have been connected to their participation in the pathophysiology of TCIRG1-related illnesses such as osteopetrosis and congenital neutropenia. Mutations (G405R, R444L, and D517N) reported in our study are already associated with osteopetrosis. These three mutations are predicated deleterious by all of the algorithm tools except one tool while ConSurf show that they are highly conserved. Our study confirms that these three mutations are shown to destabilize the TCIRG1 protein structure and function. Mutation V52L was identified by analyzing whole genome sequencing data of the patient suspected for congenital neutropenia and was carried out using the BWA, GATK4, VCFtools software. This mutation is shown by our study deleterious, highly conserved and destabilizing the protein structure and function. Fifteen nsSNPs (S474W, G458S, R56P, G379S, R757C, N730S, V375M, T314M, R92W, T368M, A417T, R363C, R56W, A778V and R50C) reported in our study are also deleterious they are not shown damaging by one or two predication tools but they also showed to destabilize protein stability and might be important nSNPs for TCIRG1 gene. ConSurf uses a combination of evolutionary conservation data and solvent accessibility predictions to determine whether an amino acid is conserved, exposed, functional, or structural. Highly conserved residues are predicted to be structurally or functionally relevant based on their positions on the protein surface and core (Ashkenazy et al., 2016). Amino acids involved in protein–protein interactions, for example, are expected to be more conserved. As a result, the nsSNPs that have been found in conserved areas are the most damaging nsSNPs (Miller and Kumar, 2001). Only 26 SNPs out of a total of 56 nsSNPs are found at evolutionary conserved, exposed, and functionally relevant residues (A20V, R56P, R57H, R191H, G192C, E321K, R366H, T368M, R444L, and E722K). There were 16 nsSNPs found at conserved, buried, and structurally significant residues (S7K, V52L, G379S, M403I, G405R, G458S, D517N, F529L, S532C, M546V, A640S, D683H, I732N, N730S, A732T, and H804Q). The remaining nsSNPs were discovered in either exposed or buried residues that were not predicted to have any structural or functional significance in the TCIRG1 protein. The I-Mutant 3.0 web server was used to estimate protein stability, and variations T570M, P572L, M546V, I721N, F610S, A732T, F51S, A717T, R57H, R109W, R191H, G192S, F529L, G458W, R444L, R56P, G379S, N730S, V375M, R92W, and T368 All of these nsSNPs can be important in the diagnosis of the TCIRG1 gene because they reduce the protein’s stability. In silico tools have been used to conduct various investigations on genes and proteins such as the CCBE1, ADA, and GJA3 genes (Shinwari et al., 2021; Essadssi et al., 2019; Zhang et al., 2020). Such research may lead to the discovery of novel therapeutic targets. All of the simulated structures were validated using RAMPAGE data. Protein designs with core RAMPAGE values greater than 80% are regarded to be superior (Essadssi et al., 2019). RAMPAGE values for the structure shown in Figure 5A (TCGIR1 wild type) were 90.5% preferred residues, 8.8% allowed, 0.6% usually allowed, and 0.2% forbidden. Similarly, for mutants P572L (90.7% favored residues, 8.6% allowed, 0.5% generally allowed, and disallowed 0.2%), R92W (90.5% favored residues, 8.8% allowed, 0.6% generally allowed, and disallowed 0.2%) R444L (90.6% favored residues, 8.8% allowed, 0.3% generally allowed, and disallowed 0.2%), and N730I (90.4% favored residues, 8.8% allowed, 0.3% generally allowed, and disallowed 0.3% and S532C (90.2% favored residues, 8.8% allowed, 0.5% generally allowed, and disallowed 0.6%, and A732T (90.6% favored residues, 8.4% allowed, 0.9% generally allowed, and disallowed 0.2% all the structures were somehow validated. Protein shapes and functions are influenced by PTMs, which have been found to be important in cell signaling, protein–protein interactions, and other essential events in biological systems (Dai and Gu, 2010; Shiloh and Ziv, 2013). We wanted to determine if the selected nsSNPs changed the PTMs of the TCIRG1 protein in this investigation. PTM sites in the protein under research were predicted using a variety of bioinformatics methods. Because lysine residues in certain proteins are methylated, this changes their interaction with DNA and regulates gene expression, methylation is a key PTM. Another essential method for protein regulation is the molecular switch, which adapts the protein to execute functions such as protein structure conformational changes, protein activation and deactivation, and signal transduction pathways (Deutscher and Saier, 2005; Puttick et al., 2008; Cieśla et al., 2011; Sawicka and Seiser, 2014). Among these predictions, the ConSurf Conservation profile shows that rs137 6162684 is highly conserved, exposed, and functionally relevant, indicating its relevance. Phosphorylation capability is demonstrated at position rs137 6162684, which also happens to be structurally essential and highly conserved (ConSurf Prediction), making it incredibly crucial. Ubiquitylation is a protein degradation mechanism that also helps to repair DNA damage (Gallo et al., 2017). Protein function and stability are both dependent on it. In protein–protein interactions, it has a structural role. As revealed by these PTM predictions, phosphorylation is the only PTM that may have a significant impact on TCIRG1 protein structure and function, with residuals rs121908251 and other reported locations in our study having the most significant phosphorylation sites. All of the phosphorylation and ubiquitylation sites identified in our investigation could play a significant role in protein stability and other TCIRG1 gene-related functions. According to GeneMANIA’s predictions, TCIRG1 is the most interacting gene in our study and co-expressed with a variety of genes. Any of the most detrimental nsSNPs in the TCIRG1 gene will eventually influence and impair the normal functioning of other linked genes, based on their interaction patterns and coexpression profiles. This highlights the significance of these interconnected and co-expressed genes in congenital neutropenia and other primary immunodeficiency disorders. Our research has all of the essential data and analyses for finding the most damaging nsSNPs because it was thorough. Every study, including ours, is limited in some way. Our research is centered on computer tools and web servers that use mathematical and statistical methodologies. As a result, further research is needed to corroborate these findings. Our findings shed light on TCIRG1 nsSNPs, their conservation, impact on protein stability and functions, protein 3D structure, PTM potential sites, ligand binding sites, and gene-gene interactions with other genes, all of which could be useful in future TCIRG1 research to better understand its role in diseases such as osteopetrosis and congenital neutropenia. The effect of substitutions on protein function was investigated using FTSite. Three ligand-binding sites were predicted by the FTSite server, each having 14.9 and 13 residues. We discovered that several alterations are involved in the ligand-binding region and form the catalytic coordination sphere, which could affect the binding affinity of the TCIRG1 protein. As predicted by SIFT software and other prediction approaches, these changes had an impact on the TCIRG1 structure and decreased its stability.

**TABLE 4:** confirmation of SIFT and Poly Phen2 predicated highly pathogenic nsSNPs through different predication tools.

Aas	LRT	Mutation taster	Mutation accessor	PROVEAN	FATHMM	VEST3	MTA SVM	METALR	M-CAP	CADD	DANN	FATHMM-MKK	PhD-SNP	PANTHER	SNP-GO	P-MUT	SNAP2
P572L	D	D	H	D	D	D	D	D	D	D	D	D	D	D	D	D	D
M546V	D	D	H	D	D	D	D	D	D	D	D	D	D	D	D	D	D
I721N	D	D	H	D	D	D	D	D	D	D	D	D	D	D	D	D	D
F610S	D	D	M	D	D	D	D	D	D	D	D	D	D	D	D	D	D
A732T	D	D	H	D	D	D	D	D	D	D	D	D	D	D	D	D	D
F51S	D	D	M	D	D	D	D	D	D	D	D	D	D	D	D	D	D
A717D	N	D	M	D	D	D	D	D	D	D	D	D	D	D	D	D	D
E722K	D	D	H	D	D	D	D	D	D	D	D	D	D	D	D	D	D
R57H	D	D	H	D	D	D	D	D	D	D	D	D	D	D	D	D	D
R109W	D	D	M	D	D	D	D	D	D	D	D	D	D	D	D	D	D
R191H	D	D	H	D	D	D	D	D	D	D	D	D	D	D	D	D	D
S532C	D	D	H	D	D	D	D	D	D	D	T	D	D	D	D	D	D
G192S	D	D	H	D	D	D	D	D	D	D	D	D	D	D	D	D	D
F529L	D	D	M	D	D	D	D	D	D	D	D	D	D	D	D	D	D
H804Q	D	D	M	D	D	D	D	D	D	D	D	D	D	D	D	D	D
G405R	D	D	-	D	D	D	D	D	D	D	D	D	D	D	D	D	D
S474W	D	D	-	D	D	D	D	D	D	D	D	D	D	D	D	D	D
G458S	D	D	-	D	D	D	D	D	D	D	D	D	D	D	D	D	D
R444L	D	D	-	D	D	D	D	D	D	D	D	D	D	D	D	D	D
R56P	D	D	-	D	D	D	D	D	D	D	D	D	D	D	D	D	D
G379S	D	D	-	D	D	D	D	D	D	D	D	D	D	D	D	D	D
R757C	D	D	M	D	D	D	D	D	D	D	T	D	D	D	D	D	D
N730S	D	D	M	D	D	T	D	D	D	D	D	D	D	D	D	D	D
V375M	D	D	-	D	D	D	D	D	D	D	D	D	D	D	D	D	D
T314M	D	D	-	D	D	D	D	D	D	D	D	D	D	D	D	D	D
D517N	D	D	H	D	D	T	D	D	D	D	D	D	D	D	D	D	D
R92W	D	D	M	D	D	T	D	D	D	D	D	D	D	D	D	D	D
T368M	D	D	-	D	D	D	D	D	D	D	D	D	D	D	D	D	D
A417T	D	D	H	D	D	D	D	D	D	D	D	T	D	D	D	D	D
R363C	D	D	-	D	D	D	D	D	D	D	D	D	D	D	D	D	D
R56W	D	D	H	D	D	T	D	T	-	D	D	D	D	D	D	D	D
A778V	D	D	M	D	D	D	D	D	D	D	D	D	D	D	N	N	N
R50C	D	D	M	D	D	T	D	D	D	D	D	D	D	D	D	D	-
V52L	D	D	M	T	D	T	D	D	-	D	D	D	D	D	D	D	D

## 5 Conclusion

Out of 64 SIFT and PolyPhen deleterious predicted nsSNPs variants, this study identified 33 novel sites which are deleterious, while 15 of which were highly deleterious variants predicted damaging/deleterious by all of the algorithms tools used in the study, and these variant mutations may lead to disruption of the original conformation of the native protein. When compared to the original protein structure, our molecular dynamics technique revealed a shift in deviation in critical locations of the mutant structures. These discrepancies can compromise the confirmation of the secondary structure and, as a result, the protein’s stability. We also noticed that the ATP binding capability of the mutant proteins was less than that of the native protein. Although the G405R, R444L, and D517N mutant has been previously associated with osteopetrosis according to the literature, no one has predicted the other 12 mutants to be linked with any diseases. As a result, it is conceivable that the unreported nsSNP can cause disease by affecting protein activation or efficiency. The findings of this study will aid future genome association studies in distinguishing harmful SNPs linked with various individual individuals with osteopetrosis and congenital neutropenia. As a result, comprehensive clinical-trial-based investigations on a broad population are required to characterize this data on SNPs, as are experimental mutational research to validate the findings.

## Data Availability

The datasets presented in this study can be found in online repositories. The names of the repository/repositories and accession number(s) can be found in the article/Supplementary Material.
