# Supplementary material for: Novel Disease-Associated Missense Single-Nucleotide Polymorphisms Variants Predication by Algorithms Tools and Molecular Dynamics Simulation of Human TCIRG1 Gene Causing Congenital Neutropenia and Osteopetrosis
Source: Front Mol Biosci. 2022 Apr 28;9:879875. doi: 10.3389/fmolb.2022.879875 (PMC9095858; doi:10.3389/fmolb.2022.879875)
Supplement: Supplementary file 1 [file Table1.DOCX]

S1Table: Overall nsSNPs sift predication in TCIRG1 gene

| ID of nsSNPs | AA Position | SIFT | Score | PolyPhen | Score |
| --- | --- | --- | --- | --- | --- |
| rs61730880 | T354M | Deleterious | 0.001 | - | - |
| rs61730880 | T570M | Deleterious | 0.002 | Probably damaging | 0.994 |
| rs115854062 | P356L | Deleterious | 0 | - | - |
| rs118141250 | V52L | Deleterious | 0.11 | Probably damaging | 0.924 |
| rs137853150 | G189R | Deleterious | 0 | - | - |
| rs137853151 | R228L | Deleterious | 0 | - | - |
| rs139397145 | R28W | Deleterious | 0 | Probably damaging | 0.967 |
| rs368945298 | M330V | Deleterious | 0 | - | - |
| rs140191063 | M403I | Deleterious | 0 | Probably damaging | 1 |
| rs140191063 | M187I | Deleterious | 0 | - | - |
| rs140963213 | A201T | Deleterious | 0.002 | - | - |
| rs144775787 | A778V | Deleterious | 0.46 | Probably damaging | 0.883 |
| rs145080707 | R213W | Deleterious  Low | 0.012 | Probably damaging | 1 |
| rs146023337 | V132M | Deleterious | 0.045 | - | - |
| rs146023337 | V348M | Deleterious | 0.048 | Probably damaging | 0.999 |
| rs148619884 | R366C | Deleterious | 0 | Benine | 0.164 |
| rs148619884 | R150C | Deleterious | 0 | - | - |
| rs371004297 | G163S | Deleterious | 0.01 | - | - |

| rs150260808 | I505N | Deleterious | 0 | - | - |
| --- | --- | --- | --- | --- | --- |
| rs150260808 | I22N | Deleterious | 0 | - | - |
| rs150788130 | A57V | Deleterious  Low | 0.006 | - | - |
| rs200209146 | N31S | Deleterious | 0.004 | Probably damaging | - |
| rs151180675 | P202L | Deleterious  Low | 0.012 | - | - |
| rs199914625 | S258W | Deleterious | 0 | - | - |
| rs200851583 | G242S | Deleterious | 0 |  |  |
| rs369983011 | R366H | Deleterious | 0.001 | Probably damaging | 0.998 |
| rs369983011 | R150H | Deleterious | 0.001 |  |  |
| rs370319355 | R50C | Deleterious | 0 | Probably damaging | 1 |
| rs371505143 | V328M | Deleterious | 0.031 | Probably damaging | 1 |
| rs372690969 | E105K | Deleterious | 0.001 | Probably damaging | 1 |
| rs372690969 | E321K | Deleterious | 0.002 | Probably damaging | 1 |
| rs372690969 | E154K | Deleterious  Low | 0.021` | - | - |
| rs372826788 | T201M | Deleterious | 0 | Probably damaging | 1 |
| rs372826788 | T43M | Deleterious  Low | 0 | - | - |
| rs375809635 | R147C | Deleterious | 0 | - | - |
| rs138305091 | A33T | Deleterious | 0 | - | - |
| rs138305091 | A516T | Deleterious | 0.001 | - | - |
| rs138308753 | F51S | Deleterious | 0 | Probably damaging | 0.996 |
| rs141095902 | A501D | Deleterious | 0.003 | - | - |
| rs141095902 | A18D | Deleterious | 0.017 | - | - |
| rs142606750 | R541C | Deleterious | 0.007 | - | - |
| rs200209146 | N514S | Deleterious | 0.024 | - | - |

| ID of nsSNPs | AA Position | SIFT | Score | PolyPhen | Score |
| --- | --- | --- | --- | --- | --- |
| rs145538370 | A424S | Deleterious | 0.023 | - | - |
| rs145538370 | A640S | Deleterious | 0.028 | Probably damaging | 0.0948 |
| rs147580611 | F394S | Deleterious | 0.001 | - | - |
| rs148921764 | E23K | Deleterious | 0 | - | - |
| rs191388932 | R183H | Deleterious  Low | 0 | Probably damaging | 1.00 |
| rs148921764 | E506K | Deleterious | 0 | - | - |
| rs367703865 | R24H | Deleterious  Low | 0 | - | - |
| rs371214361 | S316C | Deleterious | 0.001 | - | - |
| rs371658110 | G25C | Deleterious  Low | 0 | - | - |
| rs200415611 | V159M | Deleterious | 0.032 | - | - |
| rs200415611 | R178H | Deleterious  Low | 0.04 | - | - |
| rs201172503 | S173L | Deleterious  Low | 0.004 | - | - |
| rs201172503 | S129L | Deleterious | 0.041 | - | - |
| rs201172503 | S340L | Deleterious | 0.045 | Probably damaging | 0.987 |
|  |  |  |  |  |  |
| rs201661352 | R412Q | Deleterious | 0.005 | - | - |
| rs201661352 | R628Q | Deleterious | 0.01 | Probably damaging | 0.853 |
| rs201972729 | S3F | Deleterious | 0.001 | Probably damaging | 1.00 |
| rs367818260 | T98M | Deleterious | 0.003 | - | - |

Table S1 continue

| ID of nsSNPs | AA Position | SIFT | Score | PolyPhen | Score |
| --- | --- | --- | --- | --- | --- |
| rs367818260 | T147M | Deleterious  Low | 0.011 | - | - |
| rs369197157 | R412W | Deleterious | 0.003 | - | - |
| rs369197157 | R628W | Deleterious | 0.006 | Probably damaging | 0.996 |
| rs369264588 | D301N | Deleterious | 0 | - | - |
| rs370231613 | M783I | Deleterious | 0.016 | Benign | 0.019 |
| rs370231613 | M567I | Deleterious | 0.028 | - | - |
| rs370340623 | Y626S | Deleterious | 0 | Probably damaging | 0.993 |
| rs370340623 | Y410S | Deleterious | 0 | - | - |
| rs370395754 | R166T | Deleterious | 0.029 | Benign | 0.152 |
| rs370981468 | R467H | Deleterious | 0.002 | Probably damaging | 1.00 |
| rs370981468 | R251H | Deleterious | 0.002 | - | - |
| rs371004770 | D467H | Deleterious | 0.011 | - | - |
| rs371004770 | D683H | Deleterious | 0.018 | Probably damaging | 0.997 |
| rs372063415 | R325G | Deleterious  Low | 0 | - | - |

Table S1 continue

| ID of nsSNPs | AA Position | SIFT | Score | PolyPhen | Score |
| --- | --- | --- | --- | --- | --- |
|  |  |  |  |  |  |
| rs373988992 | T152M | Deleterious | 0 | Benign | 0.205 |
| rs376351835 | F313L | Deleterious | 0.01 | - | - |
| rs376883440 | R178C | Deleterious  Low | 0.019 | - | - |
| rs377034463 | A20V | Deleterious | 0.009 | Probably damaging | 0.835 |
| rs377606797 | R382H | Deleterious | 0.007 | Probably damaging | 0.995 |
| rs377606797 | R166H | Deleterious | 0.007 | Benign | 0.004 |
| rs371800878 | E45A | Deleterious  Low | 0 | Benign | 0.432 |
| rs377750765 | H588Q | Deleterious | 0 | - | - |
| rs200160870 | E7K | Deleterious | 0.027 | - | - |
