# Supplementary material for: Novel Disease-Associated Missense Single-Nucleotide Polymorphisms Variants Predication by Algorithms Tools and Molecular Dynamics Simulation of Human TCIRG1 Gene Causing Congenital Neutropenia and Osteopetrosis
Source: Front Mol Biosci. 2022 Apr 28;9:879875. doi: 10.3389/fmolb.2022.879875 (PMC9095858; doi:10.3389/fmolb.2022.879875)
Supplement: Supplementary file 2 [file Table8.DOCX]

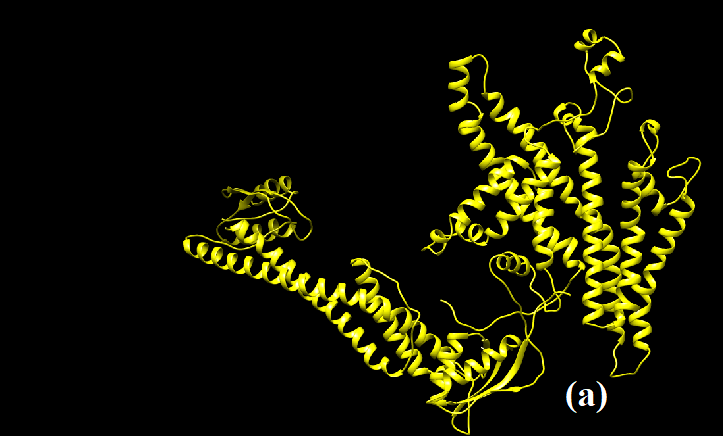

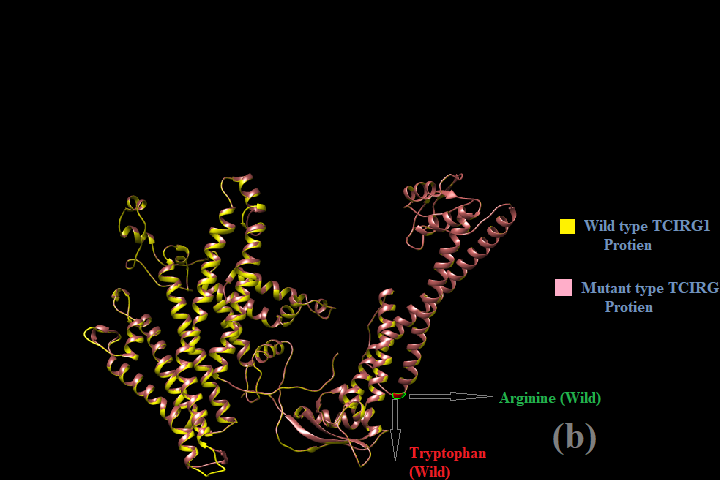


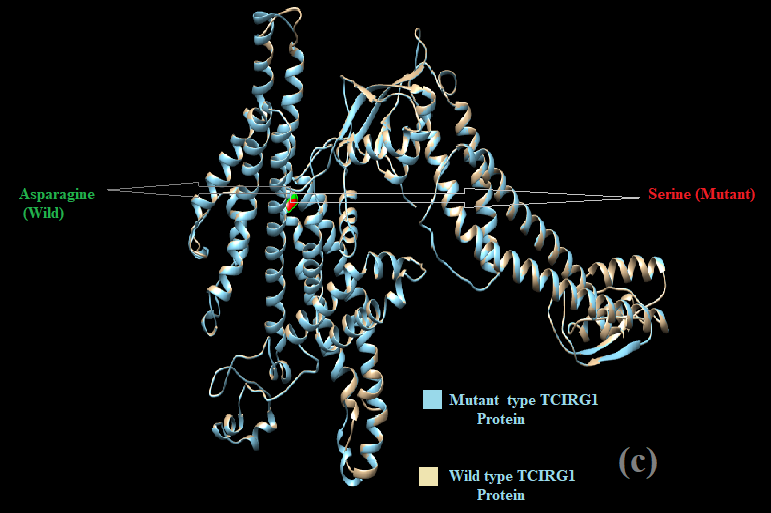

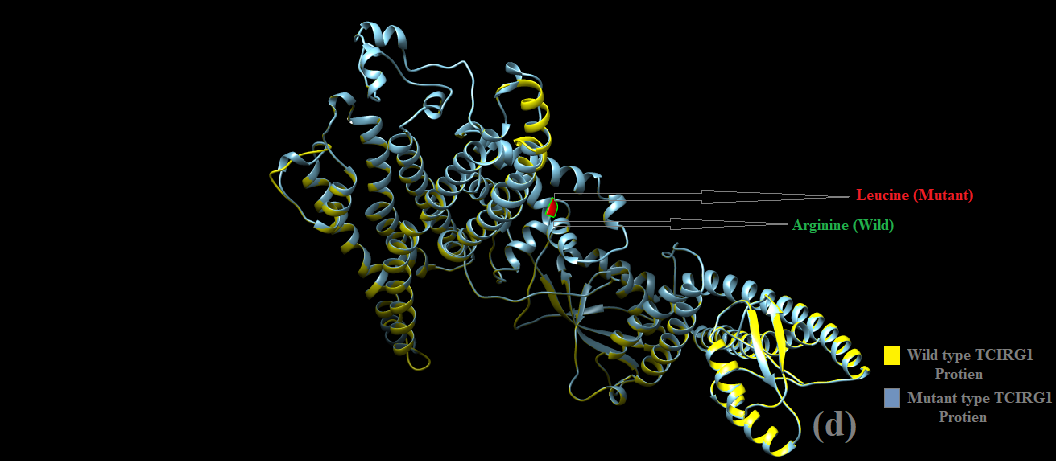


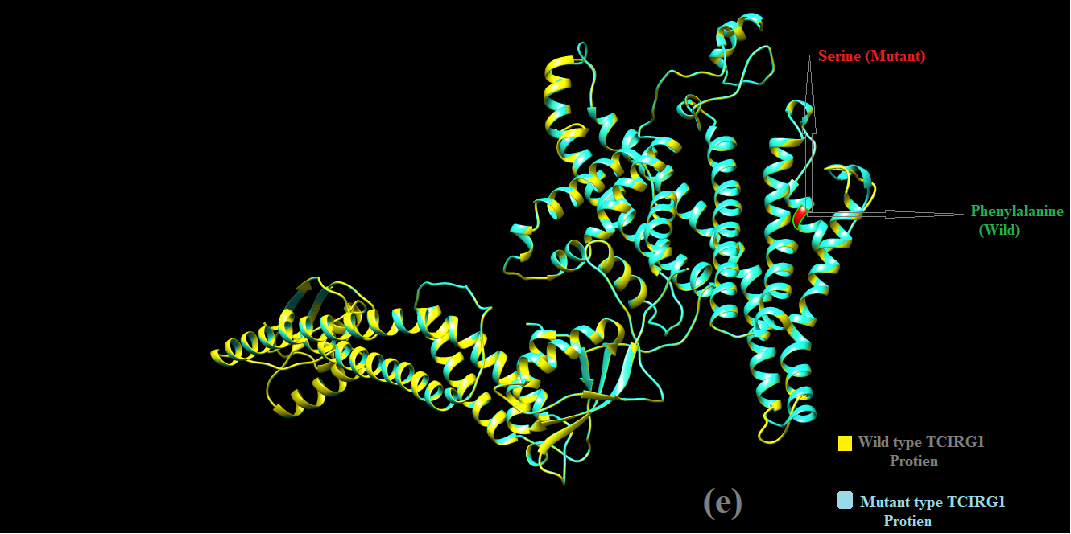


**Supplementary file 9** (a) Wild‐type TCIRG1 protein structure. (b) Superimposed structure of TCIRG1 and its R92W mutant. (c) Superimposed structure of TCIRG1 and its N730S mutant. (d) Superimposed structure of TCIRG1 and its R444L mutant (e) Superimposed structure of TCIRG1 and its F610S mutant.
