# Supplementary material for: Novel Disease-Associated Missense Single-Nucleotide Polymorphisms Variants Predication by Algorithms Tools and Molecular Dynamics Simulation of Human TCIRG1 Gene Causing Congenital Neutropenia and Osteopetrosis
Source: Front Mol Biosci. 2022 Apr 28;9:879875. doi: 10.3389/fmolb.2022.879875 (PMC9095858; doi:10.3389/fmolb.2022.879875)
Supplement: Supplementary file 3 [file Table7.DOCX]

S7 Table: Residue at ligand binding sites of TCIRG1 protein

| **Site 1** | **Site 2** | **Site 3** |
| --- | --- | --- |
| PHE X 400 | PHE X 398 | VAL/ RX0/X 26 |
| LEU X 412 | ALA X 401 | SER X 27 |
| MET X 413 | VAL X 402 | GLY X 30 |
| PHE X 416 | TYP X 456 | LYS RX1/X 298 |
| ILE X 436 | THR X 457 | ALA X 301 |
| TRP X 437 | ILE X 460 | VAL X 302 |
| PHE X 440 | TYP X 461 | LEU X 304 |
| LEU X 447 | MET X 537 | ALA X 305 |
| MET X 450 | LEU X 541 | GLN X 308 |
| LEU X 789 |  | PHR RX2/X 823 |
| VAL X 790 |  | THR X 824 |
| GLY X 793 |  | PHR X 825 |
| LEU X 794 |  | ALA X 826 |
| PHE RX2/X 797 |  |  |
