# Supplementary material for: Novel Disease-Associated Missense Single-Nucleotide Polymorphisms Variants Predication by Algorithms Tools and Molecular Dynamics Simulation of Human TCIRG1 Gene Causing Congenital Neutropenia and Osteopetrosis
Source: Front Mol Biosci. 2022 Apr 28;9:879875. doi: 10.3389/fmolb.2022.879875 (PMC9095858; doi:10.3389/fmolb.2022.879875)
Supplement: Supplementary file 5 [file Table2.DOCX]

Supplementary file2. Fig1. Overall results of various algorithm tools used in the study

Supplementary file2. Table 1. Results of various algorithm tools other than sift and PolyPhen used in the study

| AAS | LRT | Mutation Taster | Mutation Accessor | PROVEAN | FATHMM | VEST3 | MTA SVM | METALR | M-CAP | CADD | DANN | FATHMM-MKK | PhD-SNP | PANTHER | SNP-GO | P-mut | SNAP2 |
| --- | --- | --- | --- | --- | --- | --- | --- | --- | --- | --- | --- | --- | --- | --- | --- | --- | --- |
| V348M | D | D | - | T | D | T | D | D | D | D | D | D | D | D | N | N | D |
| Y626S | N | D | M | D | D | T | D | D | D | D | T | D | D | D | D | D | D |
| V52L | D | D | M | T | D | T | D | D | - | D | D | D | D | D | D | D | D |
| R28W | D | P | N | D | D | T | D | D | D | D | D | D | D | D | D | N | D |
| E321K | D | D | - | D | D | D | T | T | D | D | D | D | D | D | D | D | D |
| A640S | D | D | M | T | D | T | D | D | D | D | D | D | D | D | D | D | N |
| M403I | D | D | - | D | D | D | D | D | D | D | D | D | D | D | N | D | N |
| R183H | D | D | - | D | D | D | D | D | D | D | D | D | - | - | - | - | - |
| R467H | D | D | - | D | D | T | D | D | D | D | D | D | N | D | N | D | D |
| R366H | N | P | - | D | D | T | D | D | D | D | D | D | N | D | N | D | D |
| S340L | N | D | - | D | D | T | D | D | D | D | D | D | N | D | N | D | D |
| S3F | D | D | M | D | T | T | D | D | D | D | D | D | N | D | N | D | N |
| R382H | N | P | - | T | D | T | D | D | D | D | D | D | D | D | N | N | D |
| R628W | N | P | N | T | D | T | T | D | D | D | D | D | D | D | D | N | D |
| R628Q | N | P | N | T | D | T | T | T | D | D | D | D | D | D | D | N | D |
| V328M | N | P | L | T | D | T | T | D | D | T | T | T | - | - | - | - | - |
| R166T | N | D | M | D | D | T | T | D | D | D | T | T | D | D | N | D | N |
| E45A | D | D | M | D | D | T | D | D | D | T | T | D | - | - | - | - | - |
| T570M | N | D | L | T | D | T | D | D | D | D | D | T | N | D | N | D | N |
| M783I | N | P | N | T | D | T | T | T | D | D | T | D | D | D | D | N | N |
| D683H | N | P | L | T | D | T | T | D | D | D | T | T | D | D | D | N | N |
| A20V | N | P | L | T | T | T | T | T | D | D | D | D | D | D | D | D | N |
| E7K | N | D | L | T | D | T | T | T | D | T | T | T | D | D | D | - | - |
| A57V | - | D | - | T | D | - | - | D | - | T | D | T | - | D | - | - | - |

Threshold: Mutation Taster: <0.5 CADD: > 20 MetaLR: > 0.5 M-Cap: > 0.025 PANTHER: probably damaging time > 450my possibly damaging" (450my > time > 200my, "probably benign" (time < 200my). VEST3: > 0.5 LRT: >0.001 PROVEAN: > -2.667 FATHMM-MKK/ : <  0.5 PhDSNP: >0.5 SNP-GO: >0.5 SNAP2: −100 (fully neutral) +100 (strong effect) PON-P2: >0.5 DANN: >0.5 Mutation Assessor: > 0.65 (‐5.545 to 5.975 (higher score ‐>  more damaging)) FATHMM: > 0.453
