# Supplementary material for: Novel Disease-Associated Missense Single-Nucleotide Polymorphisms Variants Predication by Algorithms Tools and Molecular Dynamics Simulation of Human TCIRG1 Gene Causing Congenital Neutropenia and Osteopetrosis
Source: Front Mol Biosci. 2022 Apr 28;9:879875. doi: 10.3389/fmolb.2022.879875 (PMC9095858; doi:10.3389/fmolb.2022.879875)
Supplement: Supplementary file 6 [file Table5.DOCX]

**S5 Table: NetOGlyc 4.0 Results for TCIRG1 (Wild type and final selected Mutants)**

| **Position** |  | **Score** | | | |
| --- | --- | --- | --- | --- | --- |
|  | **Wild type** | **R92W** | **R444L** | **S532C** | **N730S** |
| 3 | 0.156021 | 0.13179 | 0.176848 | 0.16436 | 0.13731 |
| 7 | 0.249416 | 0.188244 | 0.247353 | 0.233018 | 0.216715 |
| 19 | 0.2667 | 0.142223 | 0.250858 | 0.24284 | 0.195603 |
| 24 | 0.0502611 | 0.0273437 | 0.0460882 | 0.0489873 | 0.0479429 |
| 27 | 0.267331 | 0.179711 | 0.228008 | 0.215532 | 0.237835 |
| 43 | 0.513032 | 0.459449 | 0.512747 | 0.511382 | 0.537428 |
| 45 | 0.325612 | 0.23252 | 0.308185 | 0.333513 | 0.347388 |
| 64 | 0.0814161 | 0.0735173 | 0.0760318 | 0.0779735 | 0.0809809 |
| 66 | 0.139722 | 0.127526 | 0.135979 | 0.13575 | 0.142113 |
| 101 | 0.460047 | 0.343066 | 0.460688 | 0.478453 | 0.457071 |
| 145 | 0.554065 | 0.575741 | 0.560139 | 0.54578 | 0.546298 |
| 149 | 0.884332 | 0.877015 | 0.889915 | 0.879581 | 0.881329 |
| 152 | 0.830233 | 0.833318 | 0.84063 | 0.825562 | 0.827838 |
| 197 | 0.412443 | 0.39954 | 0.405618 | 0.415033 | 0.39849 |
| 210 | 0.142538 | 0.132321 | 0.18997 | 0.218697 | 0.163839 |
| 215 | 0.101447 | 0.100986 | 0.128852 | 0.157631 | 0.120452 |
| 218 | 0.0567335 | 0.0482424 | 0.0806824 | 0.102906 | 0.0714166 |
| 222 | 0.0387009 | 0.0338723 | 0.0502908 | 0.0611313 | 0.0427842 |
| 236 | 0.245705 | 0.230418 | 0.275711 | 0.27642 | 0.269473 |
| 264 | 0.343619 | 0.378386 | 0.36219 | 0.347995 | 0.341802 |
| 274 | 0.250756 | 0.248942 | 0.260721 | 0.259862 | 0.243542 |
| 279 | 0.159817 | 0.156098 | 0.165028 | 0.165053 | 0.154359 |
| 310 | 0.234136 | 0.238661 | 0.227397 | 0.24521 | 0.243153 |
| 312 | 0.0825503 | 0.0870163 | 0.0972477 | 0.0939846 | 0.0874817 |
| 313 | 0.0670634 | 0.0668795 | 0.0724002 | 0.0707794 | 0.0711849 |
| 314 | 0.0598493 | 0.0607201 | 0.0697126 | 0.0671086 | 0.0656953 |
| 325 | 0.0659692 | 0.0664957 | 0.0730405 | 0.0703443 | 0.0704857 |
| 339 | 0.193567 | 0.196297 | 0.192827 | 0.222102 | 0.198707 |
| 340 | 0.21701 | 0.220928 | 0.21589 | 0.250883 | 0.219545 |
| 346 | 0.585103 | 0.582909 | 0.565822 | 0.608522 | 0.588974 |
| 360 | 0.478842 | 0.5 | 0.48320 | 0.528365 | 0.5 |
| 364 | 0.248387 | 0.267777 | 0.257535 | 0.276768 | 0.262393 |
| 368 | 0.255113 | 0.259283 | 0.245893 | 0.26139 | 0.257115 |
| 370 | 0.293127 | 0.301467 | 0.288469 | 0.316094 | 0.297623 |
| 392 | 0.057748 | 0.0580035 | 0.0553367 | 0.0580525 | 0.0573377 |
| 395 | 0.0194388 | 0.0187498 | 0.0180961 | 0.0189486 | 0.0194578 |
| 439 | 0.00592157 | 0.0064089 | 0.00504495 | 0.00667699 | 0.0056665 |
| 454 | 0.00515256 | 0.00531199 | 0.00462455 | 0.00532398 | 0.00510583 |
| 457 | 0.00355476 | 0.00373541 | 0.0042992 | 0.00376927 | 0.00352233 |
| 466 | 0.0477359 | 0.0536985 | 0.0532346 | 0.0530049 | 0.0567369 |
| 469 | 0.0470171 | 0.056713 | 0.0521538 | 0.0557896 | 0.0522998 |
| 470 | 0.134533 | 0.154663 | 0.153984 | 0.150564 | 0.145127 |
| 474 | 0.511937 | 0.550592 | 0.538115 | 0.519731 | 0.56698 |
| 477 | 0.486583 | 0.505944 | 0.483234 | 0.509289 | 0.514928 |
| 485 | 0.166765 | 0.170255 | 0.145564 | 0.161583 | 0.194328 |
| 488 | 0.386045 | 0.404925 | 0.368241 | 0.352459 | 0.370637 |
| 496 | 0.183178 | 0.194632 | 0.165866 | 0.165177 | 0.166623 |
| 499 | 0.281867 | 0.316047 | 0.295634 | 0.263347 | 0.297401 |
| 505 | 0.283697 | 0.261775 | 0.218347 | 0.253045 | 0.278015 |
| 521 | 0.0256757 | 0.0209573 | 0.0182896 | 0.0281817 | 0.0327857 |
| 528 | 0.0107963 | 0.00808072 | 0.00785621 | 0.00776651 | 0.0120098 |
| 532 | 0.00664579 | 0.00475478 | 0.00478185 | Loss of Glycosylation site | 0.00643956 |
| 538 | 0.0192876 | 0.0163749 | 0.0166406 | 0.0129588 | 0.0197934 |
| 570 | 0.0716823 | 0.0739662 | 0.0799554 | 0.0607991 | 0.0716272 |
| 575 | 0.0145691 | 0.0154076 | 0.0162698 | 0.0123972 | 0.0156075 |
| 602 | 0.0395319 | 0.0416974 | 0.0435753 | 0.0503071 | 0.0409488 |
| 605 | 0.0154055 | 0.0147611 | 0.0152941 | 0.017098 | 0.0142614 |
| 617 | 0.0717103 | 0.0738692 | 0.0596556 | 0.053371 | 0.067058 |
| 619 | 0.038534 | 0.043904 | 0.0384831 | 0.0333922 | 0.067058 |
| 621 | 0.28838 | 0.300844 | 0.3144 | 0.263356 | 0.268488 |
| 635 | 0.0512219 | 0.0523891 | 0.0578501 | 0.0514917 | 0.0493958 |
| 651 | 0.049575 | 0.0488853 | 0.0472973 | 0.0475824 | 0.0451184 |
| 685 | 0.0578354 | 0.0899885 | 0.0562459 | 0.0930107 | 0.103584 |
| 690 | 0.0316293 | 0.0418935 | 0.0330898 | 0.050357 | 0.0501437 |
| 691 | 0.0371608 | 0.06241 | 0.0435473 | 0.0617987 | 0.067596 |
| 710 | 0.0151225 | 0.0169581 | 0.0161328 | 0.0200166 | 0.0260126 |
| 720 | 0.00000326325 | 3.51434e-06 | 4.13122e-06 | 5.90219e-06 | 9.85404e-06 |
| 729 | 0.00300983 | 0.00339096 | 0.0032057 | 0.0031601 | 0.00378871 |
| 730 | - | - | - | - | Gain of Glycosylation |
| 731 | 0.00000605123 | 6.36874e-06 | 6.58277e-06 | 6.11939e-06 | 1.43604e-05 |
| 733 | 0.0000119462 | 1.16357e-05 | 1.37513e-05 | 1.58851e-05 | 1.27318e-05 |
| 741 | 0.00578932 | 0.00591909 | 0.00630668 | 0.00603827 | 0.00576532 |
| 748 | 0.00000573434 | 6.17768e-06 | 5.93472e-06 | 6.0542e-06 | 7.91175e-06 |
| 784 | 0.00636398 | 0.00676902 | 0.00675251 | 0.00652245 | 0.00628792 |
| 795 | 0.0145796 | 0.014412 | 0.014669 | 0.0143386 | 0.0134546 |
| 814 | 0.159278 | 0.167527 | 0.154613 | 0.150294 | 0.164217 |
| 816 | 0.0520322 | 0.0565478 | 0.054825 | 0.0496607 | 0.0545202 |
| 821 | 0.191626 | 0.20306 | 0.198605 | 0.188596 | 0.196478 |
| 824 | 0.201985 | 0.207272 | 0.199374 | 0.188646 | 0.210082 |
| 828 | 0.0682164 | 0.0718167 | 0.0680913 | 0.0663097 | 0.0670042 |
