# Supplementary material for: Novel Disease-Associated Missense Single-Nucleotide Polymorphisms Variants Predication by Algorithms Tools and Molecular Dynamics Simulation of Human TCIRG1 Gene Causing Congenital Neutropenia and Osteopetrosis
Source: Front Mol Biosci. 2022 Apr 28;9:879875. doi: 10.3389/fmolb.2022.879875 (PMC9095858; doi:10.3389/fmolb.2022.879875)
Supplement: Supplementary file 7 [file Table3.DOCX]

S3 table: I-Mutant 3.0 results of TCIRG1 gene highly deleterious nsSNPs

| **AAS** | **Confidence Score** | **Stability** |
| --- | --- | --- |
| P572L | -0.35 | Decrease |
| M546V | -0.56 | Decrease |
| I730N | -1.74 | Decrease |
| F610S | -1.43 | Decrease |
| A732T | -0.69 | Decrease |
| F51S | -1.78 | Decrease |
| A717D | -0.51 | Decrease |
| E722K | -0.44 | Decrease |
| R57H | -1.47 | Decrease |
| R109W | -0.06 | Decrease |
| R191W | -0.37 | Decrease |
| S532C | -0.58 | Decrease |
| G192S | -1.00 | Decrease |
| F529L | -0.95 | Decrease |
| H804Q | -0.10 | Decrease |
| G405R | -0.28 | Increase |
| S474W | -0.10 | Increase |
| G458S | -1.26 | Decrease |
| R444L | -0.23 | Decrease |
| R56P | -0.85 | Decrease |
| G379S | -1.41 | Decrease |
| R757C | -1.00 | Decrease |
| N730S | -0.34 | Decrease |
| V375M | -1.06 | Decrease |
| T314M | 0.02 | Decrease |
| D517N | -0.98 | Decrease |
| R92W | -0.24 | Decrease |
| T368M | -0.37 | Decrease |
| A417T | -0.78 | Decrease |
| R363C | -1.00 | Decrease |
| R56W | -0.49 | Decrease |
| A778V | -0.15 | Increase |
| R50C | -1.20 | Decrease |
|  |  |  |
