# Supplementary material for: Novel Disease-Associated Missense Single-Nucleotide Polymorphisms Variants Predication by Algorithms Tools and Molecular Dynamics Simulation of Human TCIRG1 Gene Causing Congenital Neutropenia and Osteopetrosis
Source: Front Mol Biosci. 2022 Apr 28;9:879875. doi: 10.3389/fmolb.2022.879875 (PMC9095858; doi:10.3389/fmolb.2022.879875)
Supplement: Supplementary file 8 [file Table4.DOCX]

**S4 Table:** Prediction of Phosphorylation Sites by NetPhos 3.1 and GPS 3.0

|  | NetPhos 3.1 | | | GPS 3.0 | | | |
| --- | --- | --- | --- | --- | --- | --- | --- |
|  | Position | Score | Kinase | Position | Score | Cutoff | Kinase |
| Serine (S) | 3 | 0.568 | PKC | 3* | 5.098 | 2.819 | AGC/DMPK/GEK/DMPK |
|  | 7 | 0.572 | unsp | 7* | 4.198 | 2.819 | AGC/DMPK/GEK/DMPK |
|  | 27 | 0.469 | CaM-II | 27* | 11.634 | 9.012 | AGC/RSK |
|  | 43 | 0.638 | PKC | 43* | 0.001 | 0.001 | AGC/NDR |
|  | 45 | 0.436 | CaM-II | 45* | 9.251 | 9.021 | AGC/RSK |
|  | 149 | 0.445 | CaM-II | 149* | 0.002 | 0.001 | AGC/NDR |
|  | 197 | 0.782 | unsp | 197* | 4.668 | 4.14 | AGC/PDK1/PDPK1 |
|  | 279 | 0.954 | unsp | 279* | 0.003 | 0.001 | AGC/NDR/LATS/LATS2 |
|  | 310 | 0.483 | CaM-II | 310* | 5.785 | 4.14 |  |
|  | 312 | 0.443 | cdc2 | 312* | 2.473 | 2.053 | AGC/RSK/RSKp90/RPS6KA1 |
|  | 325 | 0.618 | PKA | 325* | 0.003 | 0.001 | AGC/NDR/LATS/LATS1 |
|  | 339 | 0.582 | Cdk5 | 339* | 0.002 | 0.001 | AGC/NDR/LATS/LATS1 |
|  | 340 | 0.666 | Cdk5 | 340* | 0.046 | 0.007 | AGC/NDR/LATS/LATS2 |
|  | 346 | 0.707 | unsp | 346* |  |  |  |
|  | 370 | 0.562 | Cdk5 | 370* | 3.322 | 2.242 | AGC/PDK1/PKH2 |
|  | 454 | 0.880 | unsp | 454* | 1.964 | 1.833 | CAMK/CAMKL/PASK/PASK |
|  | 466 | 0.982 | unsp | 466* |  |  |  |
|  | 470 | 0.981 | unsp | 470* | 0.003 | 0.001 | AGC/NDR/LATS/LATS1 |
|  | 474 | 0.480 | GSK3 | 474* | 1.983 | 1.263 | AGC/PKA/PKACA |
|  | 477 | 0.995 | unsp | 477* | 1.413 | 1.09 | CAMK/CAMK1/MLKA |
|  | 485 | 0.945 | unsp | 485* | 2.639 | 1.686 | Atypical/PIKK/FRAP/TOR2 |
|  | 488 | 0.867 | unsp | 488* | 6.345 | 6.287 | AGC/RSK/RSKp90 |
|  | 521 | 0.982 | unsp | 521* | 0.192 | 0.143 | Atypical/PDHK/PDHK/PDK1 |
|  | 528 | 0.475 | GSK3 | 528* | 2.929 | 2.819 | AGC/DMPK/GEK/DMPK |
|  | 532 | 0.889 | PKC | 532* | 5.925 | 4.14 | AGC/PDK1/PDPK1 |
|  | 538 | 0.706 | PKA | 538 | 8.16 | 8.113 | AGC/PKG/PRKG1 |
|  | 602 | 0.639 | unsp | 602 | 0.003 | 0.001 | AGC/NDR/LATS/LATS1 |
|  | 605 | 0.448 | CaM-II | 605 | 11.056 | 10.932 | CAMK/CAMK1/CAMK4 |
|  | 617 | 0.516 | PKC | 617 | 1.419 | 1.418 | CAMK/CAMKL/QIK/SIK2 |
|  | 619 | 0.950 | unsp | 619 | 1.478 | 1.418 | CAMK/CAMKL/QIK/SIK2 |
|  | 621 | 0.479 | GSK3 | 621 | 1.456 | 1.418 | Atypical/PIKK/ATR/ATR |
|  | 685 | 0.480 | CaM-II | 685 | 0.654 | 0.638 | CAMK/CAMKL/BRSK/BRSK1 |
|  | 690 | 0.872 | unsp | 690 | 4.221 | 3.614 | CAMK/CAMKL/BRSK/BRSK2 |
|  | 691 | 0.970 | unsp |  |  |  |  |
|  | 710 | 0.469 | cdc2 | 710 | 0.276 | 0.261 | CAMK/CAMKL/MARK/MARK1 |
|  | 729 | 0.455 | CaM-II | 729 |  |  |  |
|  | 733 | 0.478 | cdc2 | 733 | 2.609 | 1.384 | Atypical/PDHK/PDHK/PDK2 |
|  | 741 | 0.494 | cdc2 | 741 | 0.001 | 0.001 | AGC/NDR/LATS/LATS1 |
|  | 748 | 0.462 | cdc2 | 748 | 2.881 | 2.862 | CAMK/CAMK-Unique/SRK2E |
|  | 795 | 0.461 | PKA | 795 | 1.87 | 1.883 | CAMK/CAMKL/PASK/PASK |
|  | 814 | 0.485 | PKA | 814 | 4.622 | 2.097 | AGC/PKC/PKCa/PRKCB |
|  | 821 | 0.561 | p38MAPK | 821 | 2.03 | 1.263 | AGC/PKA/PKACA |
| Threonine (T) | 19 | 0.526 | PKG | 19* | 9.416 | 9.293 | Atypical/PIKK/DNAPK/PRKDC |
|  | 24 | 0.446 | GSK3 | 24* | 0.199 | 0.143 | Atypical/PDHK/PDHK/PDK1 |
|  | 64 | 0.448 | CaM-II | 64* | 0.25 | 0.143 | Atypical/PDHK/PDHK/PDK1 |
|  | 66 | 0.460 | CaM-II | 66* | 0.175 | 0.143 | Atypical/PDHK/PDHK/PDK1 |
|  | 101 | 0.940 | unsp | 101* | 2.998 | 2.819 | AGC/DMPK/GEK/DMPK |
|  | 145 | 0.471 | unsp | 145* | 0.161 | 0.143 | Atypical/PDHK/PDHK/PDK1 |
|  | 152 | 0.495 | p38MAPK | 152* | 4.882 | 4.14 | AGC/PDK1/PDPK1 |
|  | 210 | 0.454 | GSK3 | 210* | 7.904 | 7.091 | CAMK/CAMKL/CHK1/CHEK1 |
|  | 215 | 0.468 | p38MAPK | 215 | 7.631 | 7.252 | CAMK/CAMKL/QIK/SIK1 |
|  | 218 | 0.552 | PKG | 218* | 0.166 | 0.143 | Atypical/PDHK/PDHK/PDK1 |
|  | 236 | 0.637 | PKG | 236* | 0.003 | 0.001 | AGC/NDR/LATS/LATS1 |
|  | 274 | 0.450 | CaM-II | 274* | 1.464 | 1.384 | Atypical/PDHK/PDHK/PDK2 |
|  | 313 | 0.704 | PKC |  |  |  |  |
|  | 314 | 0.601 | PKC | 314* | 0.467 | 0.463 | AGC/NDR/LATS |
|  | 360 | 0.590 | PKC | 360* | 0.167 | 0.143 | Atypical/PDHK/PDHK/PDK1 |
|  | 364 | 0.464 | GSK3 | 364* | 27.305 | 20.98 | AGC/DMPK/GEK |
|  | 368 | 0.536 | PKG | 368* | 8.218 | 7.383 | AGC/PKC/PKCi/PRKCI |
|  | 392 | 0.450 | CdC2 |  |  |  |  |
|  | 395 | 0.478 | PKG | 395* | 75.091 | 65.184 | AGC/PKN/PKN1 |
|  | 439 | 0.558 | PKC | 439* | 4.46 | 4.14 | AGC/PDK1/PDPK1 |
|  | 457 | 0.465 | CaM-II | 457* | 2.244 | 2.053 | AGC/RSK/RSKp90/RPS6KA1 |
|  | 469 | 0.664 | PKC |  |  |  |  |
|  | 496 | 0.489 | CaM-II | 496* | 1.5 | 1.384 | Atypical/PDHK/PDHK/PDK2 |
|  | 499 | 0.669 | PKC | 499* | 10.041 | 9.93 | Atypical/PIKK/DNAPK |
|  | 505 | 0.475 | CaM-II | 505* | 1.207 | 1.09 | CAMK/CAMK1/MLKA |
|  | 570 | 0.508 | CKII | 570* | 0.228 | 0.261 | CAMK/CAMKL/MARK/MARK1 |
|  | 575 | 0.469 | Cdc2 | 575* | 1.958 | 1.384 | Atypical/PDHK/PDHK/PDK2 |
|  | 635 | 0.453 | CaM-II | 635* | 2.298 | 2.242 | AGC/PDK1/PKH2 |
|  | 651 | 0.522 | p38MAPK | 651* | 25.84 | 20.98 | AGC/DMPK/GEK |
|  | 720 | 0.439 | CaM-II | 720* | 0.001 | 0.001 | AGC/NDR/LATS/LATS1 |
|  | 731 | 0.444 | GSK3 |  |  |  |  |
|  | 784 | 0.431 | CaM-II | 784* | 1.978 | 1.833 | CAMK/CAMKL/PASK/PASK |
|  | 816 | 0.710 | PKC | 816* | 2.168 | 1.686 | Atypical/PIKK/FRAP/TOR2 |
|  | 824 | 0.454 | GSK3 | 824* | 4.034 | 2.242 | AGC/PDK1/PKH2 |
|  | 828 | 0.621 | CKII | 828* | 5.88 | 3.164 | CAMK/CAMKL/BRSK/BRSK2 |
| Tyrosine (Y) | 23 | 0.594 | EGFR |  |  |  |  |
|  | 223 | 0.492 | INSR |  |  |  |  |
|  | 303 | 0.430 | EGFR |  |  |  |  |
|  | 378 | 0.506 | INSR |  |  |  |  |
|  | 383 | 0.619 | EGFR |  |  |  |  |
|  | 391 | 0.517 | EGFR |  |  |  |  |
|  | 445 | 0.394 | INSR |  |  |  |  |
|  | 456 | 0.466 | INSR |  |  |  |  |
|  | 461 | 0.471 | INSR |  |  |  |  |
|  | 512 | 0.414 | INSR |  |  |  |  |
|  | 583 | 0.391 | INSR |  |  |  |  |
|  | 590 | 0.358 | INSR |  |  |  |  |
|  | 626 | 0.373 | INSR |  |  |  |  |
|  | 734 | 0.385 | INSR |  |  |  |  |
|  | 813 | 0.444 | INSR |  |  |  |  |
|  | 818 | 0.900 | unsp |  |  |  |  |

*Common in both NetPhos 3.1 and GPS 3.0.


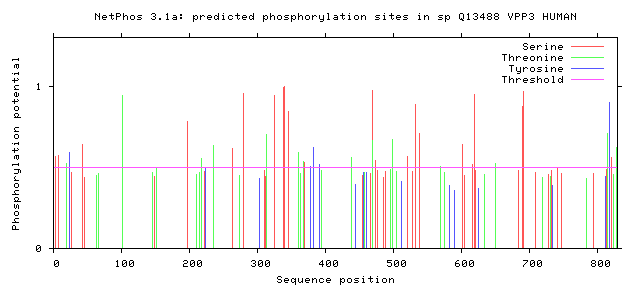


**S4 Table 2. TCIRG1 Ubiquitination Prediction Results by UbPred and BDM-PUB**

|  | **UbPred** |  | **BDM-PUB** | | |
| --- | --- | --- | --- | --- | --- |
| **Residue** | **Score** | **Ubiquitinated** | **Position** | **Score** | **Threshold** |
| 63 | 0.52 | No | 83 | 1.63 | 0.3 |
| 83 | 0.39 | No | 178 | 2.14 | 0.3 |
| 178 | 0.32 | No | 231 | 1.27 | 0.3 |
| 231 | 0.38 | No | 430 | 1.85 | 0.3 |
| 234 | 0.37 | No | 675 | 1.29 | 0.3 |
| 298 | 0.52 | No | 695 | 1.19 | 0.3 |
| 300 | 0.51 | No |  |  |  |
| 316 | 0.34 | No |  |  |  |
| 430 | 0.48 | No |  |  |  |
| 534 | 0.39 | No |  |  |  |
| 536 | 0.38 | No |  |  |  |
| 591 | 0.45 | No |  |  |  |
| 675 | 0.96 | Yes   High confidence |  |  |  |
| 695 | 0.85 | Yes   High confidence |  |  |  |
| 811 | 0.46 | No |  |  |  |
| 819 | 0.64 | Yes   Low confidence |  |  |  |

**Legend for UbPred:**

| Label | Score range | Sensitivity | Specificity |
| --- | --- | --- | --- |
| Low confidence | 0.62 ≤ s ≤ 0.69 | 0.464 | 0.903 |
| Medium confidence | 0.69 ≤ s ≤ 0.84 | 0.346 | 0.950 |
| High confidence | 0.84 ≤ s ≤ 1.00 | 0.197 | 0.989 |
