# Supplementary material for: Novel Disease-Associated Missense Single-Nucleotide Polymorphisms Variants Predication by Algorithms Tools and Molecular Dynamics Simulation of Human TCIRG1 Gene Causing Congenital Neutropenia and Osteopetrosis
Source: Front Mol Biosci. 2022 Apr 28;9:879875. doi: 10.3389/fmolb.2022.879875 (PMC9095858; doi:10.3389/fmolb.2022.879875)
Supplement: Supplementary file 9 [file Table11.DOCX]

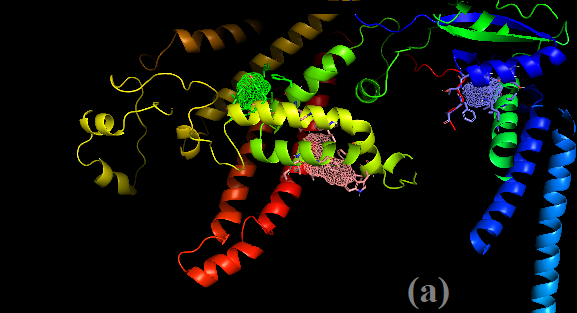


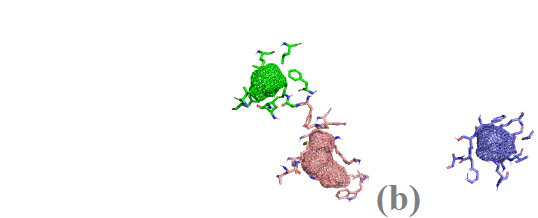


**Supplementary file 8 Fig:** (a) Ligand-binding site prediction by FT site in whole predicated 3D protein model of TCIRG1 gene (b) 3 meshes were predicted as ligand-binding site.
