# Supplementary material for: Novel Disease-Associated Missense Single-Nucleotide Polymorphisms Variants Predication by Algorithms Tools and Molecular Dynamics Simulation of Human TCIRG1 Gene Causing Congenital Neutropenia and Osteopetrosis
Source: Front Mol Biosci. 2022 Apr 28;9:879875. doi: 10.3389/fmolb.2022.879875 (PMC9095858; doi:10.3389/fmolb.2022.879875)
Supplement: Supplementary file 10 [file Table6.DOCX]

Supplementary file 6 Table: Mutprd results for the nsSNPs in TCGIR1 gene

| Genes | Mutation |  | Score |  |  | Altrations |  |
| --- | --- | --- | --- | --- | --- | --- | --- |
| VPP3_HUMAN | R56W |  | 0.591 |  |  | Altered Coiled coil (Pr = 0.87 \| P = 1.5e-03); Loss of Helix (Pr = 0.28 \| P = 0.02) | ELME000012\|ELME000102\|ELME000108 |
| VPP3_HUMAN | T570M |  | 0.527 |  |  | Altered Transmembrane protein (Pr = 0.27 \| P = 5.2e-04) | ELME000064\|ELME000220\|ELME000231\|ELME000326\|ELME000333\|ELME000336\|PS00006 |
| VPP3_HUMAN | P572L |  | 0.89 |  |  | Altered Transmembrane protein (Pr = 0.24 \| P = 1.5e-03) | ELME000045\|ELME000064\|ELME000147\|ELME000220\|ELME000231\|ELME000333\|ELME000335\|ELME000 |
| VPP3_HUMAN | G405R |  | 0.938 |  |  | Gain of Helix (Pr = 0.27 \| P = 0.04); Altered Metal binding (Pr = 0.22 \| P = 0.01); Loss of Catalytic site at D406 (Pr = 0.20 \| P = 0.01); Gain of Allosteric site at H409 (Pr = 0.19 \| P = 0.04); Altered Transmembrane protein (Pr = 0.01 \| P = 9.5e-03) | None |
| VPP3_HUMAN | R444L |  | 0.954 |  |  | Loss of Allosteric site at R444 (Pr = 0.43 \| P = 3.8e-04); Altered Ordered interface (Pr = 0.36 \| P = 2.1e-03); Altered Transmembrane protein (Pr = 0.20 \| P = 5.2e-03) | ELME000012\|ELME000045\|ELME000106\|ELME000146\|ELME000149\|ELME000231\|ELME000233 |
| VPP3_HUMAN | M403I |  | 0.924 |  |  | Altered Metal binding (Pr = 0.20 \| P = 0.02); Gain of Catalytic site at D406 (Pr = 0.18 \| P = 0.02); Altered Transmembrane protein (Pr = 0.01 \| P = 0.01) | None |
| VPP3_HUMAN | A417T |  | 0.841 |  |  | Altered Transmembrane protein (Pr = 0.01 \| P = 0.01) | ELME000336 |
| VPP3_HUMAN | A778V |  | 0.584 |  |  | Altered Transmembrane protein (Pr = 0.15 \| P = 0.01) | None |
| VPP3_HUMAN | S474W |  | 0.891 |  |  | Altered Ordered interface (Pr = 0.24 \| P = 0.04); Altered Transmembrane protein (Pr = 0.19 \| P = 7.3e-03) | ELME000063\|ELME000085\|ELME000328 |
| VPP3_HUMAN | G458S |  | 0.929 |  |  | Altered Ordered interface (Pr = 0.31 \| P = 0.01); Gain of Allosteric site at F453 (Pr = 0.22 \| P = 0.03); Altered Transmembrane protein (Pr = 0.18 \| P = 8.1e-03); Gain of Catalytic site at E463 (Pr = 0.15 \| P = 0.02); Altered Metal binding (Pr = 0.14 \| P = 0.04) | ELME000052\|ELME000053\|ELME000063\|ELME000120\|ELME000182 |
| VPP3_HUMAN | R50C |  | 0.626 |  |  | Altered Coiled coil (Pr = 0.44 \| P = 8.8e-03); Loss of Pyrrolidone carboxylic acid at Q48 (Pr = 0.04 \| P = 0.04) | ELME000012\|ELME000102\|ELME000108\|ELME000233\|ELME000300\|ELME000328 |
| VPP3_HUMAN | E321K |  | 0.795 |  |  | Loss of Catalytic site at E321 (Pr = 0.25 \| P = 4.7e-03) | None |
| VPP3_HUMAN | R363C |  | 0.578 |  |  | Loss of Relative solvent accessibility (Pr = 0.30 \| P = 8.8e-03); Altered Disordered interface (Pr = 0.28 \| P = 0.03); Altered Transmembrane protein (Pr = 0.22 \| P = 3.2e-03); Altered DNA binding (Pr = 0.20 \| P = 0.02) | ELME000053\|ELME000336 |
| VPP3_HUMAN | A732T |  | 0.543 |  |  | Altered Ordered interface (Pr = 0.25 \| P = 0.02); Loss of GPI-anchor amidation at N730 (Pr = 0.04 \| P = 7.1e-03); Gain of N-linked glycosylation at N730 (Pr = 0.01 \| P = 0.04) | ELME000052\|ELME000053\|ELME000063\|ELME000070\|PS00001 |
| VPP3_HUMAN | F51S |  | 0.892 |  |  | Altered Coiled coil (Pr = 0.36 \| P = 0.01); Altered Stability (Pr = 0.35 \| P = 4.0e-03); Loss of Pyrrolidone carboxylic acid at Q48 (Pr = 0.04 \| P = 0.05) | ELME000012\|ELME000062\|ELME000102\|ELME000108\|ELME000233\|ELME000300\|ELME000328\|ELME000 |
| VPP3_HUMAN | F610S |  | 0.938 |  |  | Altered Transmembrane protein (Pr = 0.40 \| P = 0.0e+00) | ELME000328\|ELME000336 |
| VPP3_HUMAN | R57H |  | 0.582 |  |  | Altered Coiled coil (Pr = 0.51 \| P = 6.9e-03); Loss of Helix (Pr = 0.31 \| P = 4.8e-03) | ELME000012\|ELME000102\|ELME000108 |
| VPP3_HUMAN | N730S |  | 0.617 |  |  | Altered Ordered interface (Pr = 0.25 \| P = 0.02); Loss of GPI-anchor amidation at N730 (Pr = 0.04 \| P = 7.0e-03) | ELME000053\|ELME000063\|PS00008 |
| VPP3_HUMAN | R56P |  | 0.848 |  |  | Altered Coiled coil (Pr = 0.85 \| P = 1.7e-03); Loss of Helix (Pr = 0.36 \| P = 3.5e-04) | ELME000012\|ELME000102\|ELME000108\|ELME000155 |
| VPP3_HUMAN | M546V |  | 0.929 |  |  | Gain of Strand (Pr = 0.26 \| P = 0.04); Altered Metal binding (Pr = 0.04 \| P = 0.05); Altered Transmembrane protein (Pr = 0.01 \| P = 0.01) | None |
| VPP3_HUMAN | R628W |  | 0.515 |  |  | Altered Transmembrane protein (Pr = 0.29 \| P = 1.9e-04); Loss of Helix (Pr = 0.27 \| P = 0.04); Gain of Strand (Pr = 0.26 \| P = 0.04); Loss of Pyrrolidone carboxylic acid at Q629 (Pr = 0.08 \| P = 0.02) | ELME000117\|ELME000163 |
| VPP3_HUMAN | D517N |  | 0.891 |  |  | Loss of Catalytic site at D517 (Pr = 0.29 \| P = 2.4e-03); Altered Metal binding (Pr = 0.28 \| P = 5.5e-03); Altered Transmembrane protein (Pr = 0.28 \| P = 4.0e-04); Altered Ordered interface (Pr = 0.28 \| P = 0.03); Altered Disordered interface (Pr = 0.28 \| P = 0.03); Loss of Allosteric site at W520 (Pr = 0.25 \| P = 0.02) | None |
| VPP3_HUMAN | M783I |  | 0.654 |  |  | Altered Ordered interface (Pr = 0.23 \| P = 0.05); Altered Transmembrane protein (Pr = 0.04 \| P = 5.4e-04) | ELME000041\|ELME000052 |
| VPP3_HUMAN | Y626S |  | 0.761 |  |  | Gain of Intrinsic disorder (Pr = 0.43 \| P = 5.2e-03); Loss of Strand (Pr = 0.27 \| P = 0.02); Altered Transmembrane protein (Pr = 0.26 \| P = 9.5e-04); Altered Ordered interface (Pr = 0.24 \| P = 0.05); Loss of Pyrrolidone carboxylic acid at Q629 (Pr = 0.08 \| P = 0.02); Gain of GPI-anchor amidation at N622 (Pr = 0.02 \| P = 0.02) | ELME000106\|ELME000136\|ELME000159\|ELME000163\|PS00005 |
| VPP3_HUMAN | G379S |  | 0.863 |  |  | Altered Ordered interface (Pr = 0.31 \| P = 0.01); Gain of Relative solvent accessibility (Pr = 0.31 \| P = 6.2e-03); Gain of Allosteric site at Y383 (Pr = 0.25 \| P = 0.01); Altered Metal binding (Pr = 0.21 \| P = 0.03); Altered DNA binding (Pr = 0.21 \| P = 0.02); Gain of Catalytic site at Y383 (Pr = 0.19 \| P = 0.01); Altered Transmembrane protein (Pr = 0.17 \| P = 9.7e-03) | ELME000293 |
